# Supplementary material for: Demographic, temporal, and spatial analysis of human animal bite cases in Mymensingh District, Bangladesh
Source: PLoS Negl Trop Dis. 2025 Jun 9;19(6):e0012204. doi: 10.1371/journal.pntd.0012204 (PMC12173383; doi:10.1371/journal.pntd.0012204)
Supplement: Attachment — Submitted filename: PNTD-D-24-00663_R002.pdf [file pntd.0012204.s005.pdf]

PLOS Neglected Tropical Diseases

Demographic, temporal, and spatial analysis of human animal bite cases in Mymensingh District, Bangladesh

--Manuscript Draft--

**Manuscript Number:**	PNTD-D-24-00663R1
**Full Title:**	Demographic, temporal, and spatial analysis of human animal bite cases in Mymensingh District, Bangladesh
**Short Title:**	Human animal bite cases in Mymensingh District, Bangladesh
**Article Type:**	Research Article
**Keywords:**	rabies; incidence ratio; risk map; dogs; cats; one health; targeted intervention
**Abstract:**	Objective : This study aimed to analyze the demographic, temporal, and spatial characteristics of animal bite (AB) cases in humans across 12 Upazilas within Mymensingh district of Bangladesh.<br>Methods: Retrospective hospital-based data from individual AB cases for 2022 and 2023 were collected from S.K Hospital. The dataset included information on victim demographics, bite details, vaccination information, and Rabies Immune Globulin (RIG) administration. Additionally, monthly case counts from 2016 to 2024 were sourced and analyzed to identify trends. Descriptive statistics and time series analysis using the seasonal decomposition technique were conducted. Risk maps for animal bites in 2022 and 2023 were generated using a standardized incidence ratio (SIR) approach.<br>Findings: An almost two-fold increase in the proportion of category 3 bites receiving Rabies Immune Globulin (RIG) from 3.6% in 2022 to 6.5% in 2023 was noted. Only 9.7% of bite cases in 2022 and 16.9% in 2023 received the vaccine promptly on the day of the incident. However, the majority received vaccines within the first 24 hours after being bitten. Moreover, significant seasonal patterns and annual increasing trends in AB cases were observed. Males and children under 14 years old had a higher risk of being bitten. Dogs (48.2% in 2022) and cats (52.6% in 2023) were identified as the primary animals responsible for the bites. Notably, the legs were the body part most frequently bitten. The bites risk map identified four high risk Upazilas.<br>Conclusion: There is a significant gap in ensuring timely vaccination delivery. The study results also suggest other potential improvements in healthcare practices or treatment protocols, and the need for a veterinary surveillance system.<br>Increasing AB cases highlight the need to enhance surveillance and control measures. Targeted awareness campaigns for high-risk human groups, such as males and children under 14 years of age, are essential to reduce the risk of rabies transmission. Targeted awareness campaigns tailored to high-risk groups such as males and children under 14 years of age, along with preventive programs focused on dogs are imperative. Coordinated One Health efforts among healthcare professionals, veterinarians, policymakers, and community stakeholders are crucial to effectively mitigate the incidence of AB cases, safeguarding public health and eliminate dog mediated rabies by 2030 in the region.
**Additional Information:**	
**Question**	**Response**
**Government Employee**<br><br>Are you or any of the contributing authors an employee of the United States government?<br><br>Manuscripts authored by one or more US Government employees are not copyrighted, but are licensed under a CC0	No - No authors are employees of the U.S. government.

Powered by Editorial Manager® and ProduXion Manager® from Aries Systems Corporation

Public Domain Dedication, which allows unlimited distribution and reuse of the article for any lawful purpose. This is a legal requirement for US Government employees.<br><br>This will be typeset if the manuscript is accepted for publication.	
**Financial Disclosure**<br><br>Enter a financial disclosure statement that describes the sources of funding for the work included in this submission. Review the submission guidelines for detailed requirements. View published research articles from *PLOS Neglected Tropical Diseases* for specific examples.<br><br>This statement is required for submission and **will appear in the published article** if the submission is accepted. Please make sure it is accurate.<br><br>**Funded studies**<br>Enter a statement with the following details:<br>• Initials of the authors who received each award<br>• Grant numbers awarded to each author<br>• The full name of each funder<br>• URL of each funder website<br>• Did the sponsors or funders play any role in the study design, data collection and analysis, decision to publish, or preparation of the manuscript?<br><br>Did you receive funding for this work?	The author(s) received no specific funding for this work.
**Competing Interests**<br><br>On behalf of all authors, disclose any competing interests that could be perceived to bias this work.<br><br>This statement will be typeset if the manuscript is accepted for publication.	The authors have declared that no competing interests exist

Review the instructions link below and PLOS NTDs' competing interests policy to determine what information must be disclosed at submission.	
**Data Availability**<br><br>Provide a **Data Availability Statement** in the box below. This statement should detail where the data used in this submission can be accessed. This statement will be typeset if the manuscript is accepted for publication.<br><br>Before publication, authors are required to make all data underlying their findings fully available, without restriction. Review our PLOS Data Policy page for detailed information on this policy. Instructions for writing your Data Availability statement can be accessed via the Instructions link below.	All data generated from this study are presented within the manuscript and as supporting files.

Powered by Editorial Manager® and ProduXion Manager® from Aries Systems Corporation

Manuscript
Click here to access/download;Manuscript;Revised_Manuscript.docx

Demographic, temporal, and spatial analysis of human animal bite cases in Mymensingh District, Bangladesh

Chandra Shaker Chouhan[1#], Abu Raihan[2#], Md. Manik Mia[1#], Subarna Banerjee[3], Ishmam Shahriar[2], Progganananda Nath[4], Jasim M. Uddin [5], Md. Amimul Ehsan[1], Michael P. Ward[6], A. K. M. Anisur Rahman[1*]

[1]Department of Medicine, Faculty of Veterinary Science, Bangladesh Agricultural University, Mymensingh-2202

[2]Faculty of Veterinary Science, Bangladesh Agricultural University, Mymensingh-2202

[3]Department of Animal Science, Faculty of Animal Husbandry, Bangladesh Agricultural University, Mymensingh-2202

[4]Mymensingh Medical College and Hospital (MMCH), Mymensingh, Bangladesh

[5]School of Veterinary Medicine, Murdoch University, Perth, WA 6150, Australia

[6]School of Veterinary Science, Faculty of Science, The University of Sydney, Camden, Australia

# equally contributed

[*]**Corresponding author:** Professor Dr. A. K. M. Anisur Rahman, Department of Medicine, Faculty of Veterinary Science, Bangladesh Agricultural University, Mymensingh-2202.

Email: arahman_med@bau.edu.bd

Abstract

Objective : This study aimed to analyze the demographic, temporal, and spatial characteristics of animal bite (AB) cases in humans across 12 Upazilas within Mymensingh district of Bangladesh.

Methods: Retrospective hospital-based data from individual AB cases for 2022 and 2023 were collected from S.K Hospital. The dataset included information on victim demographics, bite details, vaccination information, and Rabies Immune Globulin (RIG) administration. Additionally, monthly case counts from 2016 to 2024 were sourced and analyzed to identify trends. Descriptive statistics and time series analysis using the seasonal decomposition technique were conducted. Risk maps for animal bites in 2022 and 2023 were generated using a standardized incidence ratio (SIR) approach.

Findings: An almost two-fold increase in the proportion of category 3 bites receiving Rabies Immune Globulin (RIG) from 3.6% in 2022 to 6.5% in 2023 was noted. Only 9.7% of bite cases in 2022 and 16.9% in 2023 received the vaccine promptly on the day of the incident. However, the majority received vaccines within the first 24 hours after being bitten. Moreover, significant seasonal patterns and annual increasing trends in AB cases were observed. Males and children under 14 years old had a higher risk of being bitten. Dogs (48.2% in 2022) and cats (52.6% in 2023) were identified as the primary animals responsible for the bites. Notably, the legs were the body part most frequently bitten. The bites risk map identified four high risk Upazilas.

Conclusion: There is a significant gap in ensuring timely vaccination delivery. The study results also suggest other potential improvements in healthcare practices or treatment protocols, and the need for a veterinary surveillance system. Increasing AB cases highlight the need to enhance surveillance and control measures. Targeted awareness campaigns for high-risk human groups, such as males and children under 14 years of age, are essential to reduce the risk of rabies

transmission. Targeted awareness campaigns tailored to high-risk groups such as males and children under 14 years of age, along with preventive programs focused on dogs are imperative. Coordinated One Health efforts among healthcare professionals, veterinarians, policymakers, and community stakeholders are crucial to effectively mitigate the incidence of AB cases, safeguarding public health and eliminate dog mediated rabies by 2030 in the region.

Key words: Rabies, incidence ratio, risk map, dogs, cats, One Health, targeted intervention

Author Summary

This study analyzed retrospective hospital-based data from individual AB cases for 2022 and 2023, as well as monthly case counts from 2016 to 2024, at S.K Hospital in Mymensingh. We observed a notable increase in severe bites (category 3) treated with Rabies Immune Globulin (RIG), rising from 3.6% in 2022 to 6.5% in 2023. Although only a small percentage of victims received vaccines on the same day as the bite (9.7% in 2022 and 16.9% in 2023), most were vaccinated within 24 hours. The study revealed seasonal patterns and a yearly increase in AB cases, with males and children under 14 being the most affected. Dogs and cats were the primary animals responsible for bites, and legs were the most frequently bitten body part. Four Upazilas were identified as high-risk areas. The findings emphasize the need for improved timely vaccination and healthcare practices. To reduce rabies risk, targeted awareness campaigns for high-risk groups such as males and children under 14, along with dog-focused prevention programs, are necessary. Collaboration among healthcare workers, veterinarians, policymakers, and communities is vital to control AB cases and eliminate dog-mediated rabies by 2030.

1. Introduction

Rabies, caused by Lyssavirus type 1 of the Rhabdoviridae family, is a fatal viral infection of the central nervous system that results in progressive encephalomyelitis (1-3). The virus is typically

present in the saliva of clinically ill mammals, which can then be transmitted to other mammals (including humans) through a bite, scratch, or lick (4). Rabies manifests in two epidemiological conditions: urban, primarily transmitted by dogs or less commonly by cats; and sylvatic, carried by wolves, foxes, raccoons, weasels, and bats (5).

In humans, transmission mainly occurs from canines, as well as from cats, mongoose, bats, and in rare cases, farm animals (6). Globally, more than 1.4 billion people are at risk of rabies, and approximately 45% of rabies-related deaths occur in Asia (7). Human rabies cases that result from dog bites constitute 97% of all cases (8). Bangladesh ranks third after India and China in terms of rabies mortalities, with more than 2100 people dying annually (9). Dogs (90%), cats (6%), jackals (3%), and mongoose (1%) were reported to be responsible for human bites in Bangladesh (9).

The most effective strategy for preventing human rabies is to target the animal reservoir, using mass dog vaccination to stop transmission (10). The World Health Organization (WHO) and its partners, including the World Organization for Animal Health (O[illegible]), the Food and Agriculture Organization of the United Nations (FAO) and the Global Alliance for Rabies Control (GARC), have adopted a goal to eliminate dog-mediated human rabies by 2030 by controlling the disease in dogs (11). In developed countries, concerted efforts including mass dog vaccination and oral vaccination of wildlife have contributed to the elimination of rabies. This has resulted in a subsequent decrease in human rabies cases, although rabies still persists in some wildlife populations (12). Nevertheless, timely wound management is crucial as an emergency medical response in disease control measures (13).

Rabies cases often do not fully represent the disease burden due to negligence and possible underreporting in specific areas (14). Unreported cases and a lack of understanding of epidemiological trends pose a significant threat to the efficient execution of preventive and control

measures (15, 16). In Bangladesh, rabies post-exposure prophylaxis (PEP) and surveillance are managed through a network of national and local health institutions. The National Rabies Prevention and Control Centre (NRPCC) in Dhaka, established in 2011, serves as the primary referral center and provides free vaccination and treatment. The Bangladesh Institute of Tropical and Infectious Diseases in Chittagong acts as the secondary referral center. Additionally, 66 public District Rabies Prevention and Control Centers offer free PEP (17). In addition to this, Bangladesh established over 300 sophisticated Animal Bite Management Centers at the sub-district (Upazila) level to prevent rabies from animal bites. A common misconception prevents proper documentation of epidemiological data on animal bite cases and fatalities. About 59% of dog bite victims initially seek treatment from traditional healers rather than visiting hospitals. Additionally, only 29% receive the rabies vaccine, 2% practice proper wound washing with soap and water, while 4.8% do not take any measures (18). Furthermore, limited financial resources impede the conduct of surveys to determine rabies incidence in animals. The lack of laboratory confirmation in the identification of animals, primarily due to financial and logistical limitations, further exacerbates the issues (19).

Implementing measures to decrease animal bite incidence is one of the critical approaches to controlling rabies (2). The Government of Bangladesh is actively working toward the Zero by 30 goal through the National Rabies Elimination Program, launched in 2011. Key rabies control strategies include advocacy, communication, and social mobilization to raise awareness, providing modern treatment for dog bites through updated post-exposure prophylaxis, conducting mass dog vaccination campaigns to reduce rabies transmission, and implementing humane dog population management (15, 17). Since 2011, these initiatives have resulted in a decline in human rabies-related fatalities. A retrospective study conducted from 2006 to 2018 in Bangladesh revealed that

each district experienced between one and ten human rabies cases. The highest number of cases recorded in any district during this period was between 41 and 60 (15). Understanding the demographic, spatial and temporal patterns of rabies in humans is crucial for assessing risks and developing targeted interventions. To do so, the current study investigated the demographic, temporal, and spatial characteristics of human animal bite (AB) cases in the Mymensingh district of Bangladesh.

2. Methodology

2.1. Study design and data source

A retrospective hospital-based cross-sectional study was conducted to collect secondary data on cases of animal bites that occurred from January 2022 to December 2023. The data were retrieved from registers maintained at the Government S.K. Hospital in Mymensingh District. The data included victim's address, age, sex, location of the bite, type of animal involved, date of the attack, date of vaccination, and details on the amount of HRIG provided, as well as the injury category. Additionally, we collected monthly total number of cases from 2016 to 2024 to assess the seasonal patterns and yearly trends in animal bite cases. No data were available for the year 2020 due to the COVID-19 pandemic.

2.2. Data processing and descriptive analysis

The data for each bite case were initially entered into Microsoft Excel 2017. Subsequently, the data were imported into Jupyter Notebook, Python, for further analysis. Descriptive statistics for continuous variables, such as age and the amount of Rabies Immune Globulin (RIG) administered, were generated using the Pandas library's 'description ()' command in Python. For categorical variables, frequency distributions were created using the Pandas library's 'value counts ()'

command. The proportion of each category for categorical variables was calculated by using the Pandas library’s ‘value_counts ()’ command with the ‘normalize=True’ parameter.

2.3. Temporal distribution analysis

We obtained monthly animal bite case data from January 2016 to December 2024. Due to incomplete data in 2020, we used the ‘forecast::na.interp()’ function in R 4.4.1 to impute missing values. This hybrid interpolation method combines seasonal-trend decomposition (STL) with linear interpolation, ensuring continuity and minimizing bias from artificial discontinuities.

The method ensures continuity in the time series while minimizing bias from artificial discontinuities, making it particularly suitable for datasets with inherent seasonality. The dataset was imported and processed in R 4.4.1. The date variable was converted to `Date` format and the dataset was arranged in chronological order to maintain temporal consistency. We transformed the monthly animal bite counts into a time series object using the `ts()` function within the package `forecast` in R. The time series spanned from January 2016 to December 2024, with a frequency of 12 to represent monthly observations. To investigate the underlying components of the time series, we performed a seasonal-trend decomposition using the LOESS (STL) method. This approach decomposes the observed series into three main components: seasonal, trend, and remainder (irregular) components. We used a periodic seasonal window (`s.window = ″periodic”`) to capture recurring seasonal patterns over the study period. We visualized the decomposition using the `autoplot()` function from the `forecast` package, which provides a clear representation of the seasonal, trend, and remainder components. The monthly animal bite cases data and the R code utilized for time series analysis are provided as supporting files 1 and 2, respectively.

2.4. Animal bite risk mapping

A standardized risk ratio map was created using the following steps in Python:

i. Global incidence calculation

The global incidence of animal bites, representing the total number of cases divided by the total population across all Upazilas, was estimated using the following formula:

$$Global\ Incidence = \frac{Total\ number\ of\ cases}{Total\ population}$$

ii. Expected number of cases for each Upazila

The expected number of cases for each Upazila was calculated by multiplying the global incidence and the population of each Upazila as shown in equation 1:

$$Expected\ number\ of\ cases = Global\ Incidence \times Population\ of\ Upazila \quad (1)$$

iii. Standardized incidence ratio (SIR) calculation

The Standardized Incidence Ratio (SIR) was then calculated for each Upazila by dividing the reported number of AB cases by the expected number of cases in each Upazila according to previously described method (20) using equation (2).

$$SIR = \frac{Actual\ number\ of\ animal\ bite\ cases}{Expected\ number\ of\ cases} \quad (2)$$

iv. Geospatial data loading

The Bangladesh Upazila level shape file was downloaded from the GADM maps and data website (https://gadm.org/). The Geopandas library was used to read a shape file containing the geographic boundaries of Upazilas in Bangladesh.

v. Upazila selection, filtering & geospatial data merging

The Upazilas of Mymensingh district were selected and filtered to create a map of Mymensingh district. The filtered geospatial data (Upazila boundaries) and the data containing SIR values were merged based on the common Upazila names.

vi. Risk ratio map creation & annotation, and visualization

Using 'matplotlib' and 'geopandas', a choropleth map was created where the fill color represented the SIR values of each Upazila. The map was visualized with distinct colors indicating variations in standardized risk ratios. Upazila names were annotated at the centroids of their respective boundaries to enhance map readability.

3. Results

3.1. Descriptive statistics

The S. K. Hospital in the Mymensingh district documented 14,943 cases of human animal bites in 2022 and 18,998 cases in 2023. However, only 9.7% in 2022 and 16.9% in 2023 received the vaccine promptly on the day of the incident. The majority received the vaccine within the first 24 hours after being bitten **(Table 1)**

Table 1. Time gap between animal bite and anti-rabies vaccination

Year	Day 0	Day 1	Day 2	Day 3	Day 4	Day 5	≥ Day 6
2022	1449 (9.7%)	9987 (66.8%)	2458 (16.5%)	628 (4.2%)	238 (1.6%)	101 (0.7%)	82 (0.5%)
CP	9.7%)	76.5%	93.0 %	97.2 %	98.8%	99.5%	100%
2023	3209 (16.9%)	12859 (67.7%)	1994 (10.5%)	588 (3.1%)	170 (0.9%)	104 (0.5%)	74 (0.4%)
CP	16.9%	84.6%	95.1%	98.2%	99.1%	99.6%	100%

CP: Cumulative proportion

In 2022, the rabies vaccine was administered in four doses, scheduled at days 0, 3, 7, and 28. However, in 2023 the vaccination protocol was revised, with only three doses given on days 0, 3, and 21. Consequently, the proportion of individuals who failed to complete the entire vaccination course decreased from 10.3% in 2022 to 7.8% in 2023 **(Table 2).**

Table 2. Completion status of full vaccination regimen

Year	Day 0		Day 3		Day 7		Day 21		Day 28	
	Yes	No	Yes	No	Yes	No	Yes	No	Yes	No
2022	14943 (100%)	0	14906 (99.7%)	37 (0.3%)	14173 (94.9%)	770 (5.2%)	–	–	13398 (89.7%)	1545 (10.3%)
2023	18998 (100%)	0	18958 (99.8%)	40 (0.2%)	–	–	17510 (92.2%)	1488 (7.8%)	–	–

The demographic characteristics of animal bite cases are presented in **Table 3**. In 2022, there were 538 cases (3.6%) categorized as type 3 bites, which increased to 6.5% in 2023, all of which exclusively received RIG. The proportion of type 3 bites varied across age groups. It was lowest among individuals aged 25–34 years (2.9%) and highest among those aged 55–64 years (6.7%). Moreover, type 3 bites were more prevalent in males (4.1%) compared to females (2.8%). The mean age of animal bite cases was 25.4 years, with an interquartile range (IQR) of 10.0 to 37.0 years. In both 2022 and 2023 the highest percentage of bite cases was in the age group 5-14 years old, 25.17% and 25.90%, respectively. More cases were observed in males, making up 63.2% of bite cases in 2022 and 61.2% in 2023. In 2022, dogs were the most common animals involved in the bites, accounting for 48.2%, whereas in 2023 cats were the majority, making up 52.6%. Besides dogs and cats, foxes, mongoose, and monkeys were also involved in bite cases. Legs account for approximately 60% of the total bite cases in 2022 and 55% in 2023, while hands account for around 32% in 2022 and 34% in 2023. Additionally, there were reports of bites to the face and even the eye.

Table 3. Demo[illegible]phic distribution of animal bite cases reported in Mymensingh district, Bangladesh

**Variables**	**Category level**	**Number of total cases (33,941)**	
		**Year 2022 (14,943)**	**Year 2023 (18,998)**
Injury category	Category 2	14405 (96.4%)	17767 (93.5%)
	Category 3	538 (3.6%)	1231 (6.48%)
Age (Years)	0-4	1482 (9.92%)	1906 (10.03%)
	5-14	3761 (25.17%)	4921 (25.9%)
	15-24	3050 (20.41%)	4054 (21.34%)

	25-34	2200 (14.72%)	2857 (15.04%)
	35-44	1618 (10.82%)	2071 (10.9%)
	45-54	1442 (9.66%)	1634 (8.6%)
	55-64	868 (5.81%)	1052 (5.54%)
	> 65	522 (3.49%)	503 (2.65%)
Sex	Male	9448 (63.2%)	11627 (61.2%)
	Female	5495 (36.8%)	7371 (38.8%)
Animals involved	Dog	7196 (48.2%)	8132 (42.8%)
	Cat	7107 (47.6%)	9983 (52.6%)
	Fox	404 (2.7%)	560 (3.0%)
	Monkey	193 (1.3%)	255 (1.3%)
	Mongoose	43 (0.3%)	68 (0.4%)
Biting sites	Right Leg	4708 (31.5%)	5863 (30.9%)
	Left Leg	4255 (28.5%)	4818 (25.4%)
	Right Hand	2467 (16.5%)	3406 (17.9%)
	Left Hand	2243 (15.0%)	3176 (16.7%)
	Thigh	281 (1.9%)	429 (2.3%)
	Back	262 (1.8%)	376 (2.0%)
	Hip	245 (1.6%)	266 (1.4%)
	Face	182 (1.2%)	253 (1.3%)
	Abdomen	81 (0.54%)	93 (0.49%)
	Both hand	61 (0.41%)	72 (0.38%)
	Both Leg	52 (0.34%)	59 (0.31%)
	Head	49 (0.33%)	61 (0.32%)
	Neck	29 (0.19%)	85 (0.45%)
	Thorax	18 (0.12%)	21 (0.11%)
	Eye	11 (0.07%)	19. (0.1%)

3.2. Month-wise distribution of animal bite cases

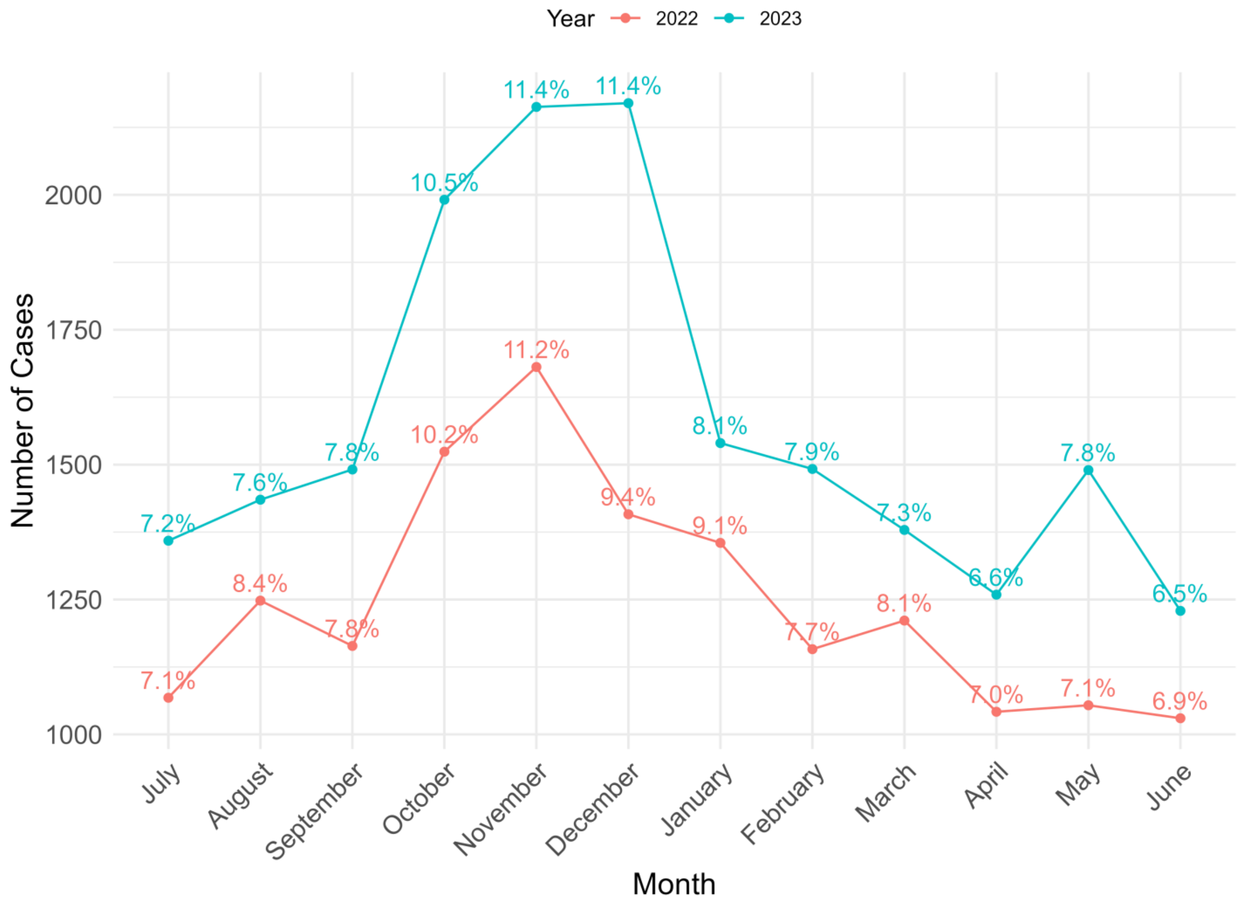

Figure 1: Month wise distribution of animal bite cases in 2022 and 2023

The monthly distribution of animal bite cases is shown in **Figure 1**. The lowest number of cases was observed in June in both 2022 (6.9%) and 2023 (6.5%), while the highest number of cases was reported in November in both 2022 (11.2%) and 2023 (11.4%). From October to December in 2022, there were 33.3% of total cases, and in 2023, this figure was 30.8%.

3.3. Spatial distribution of animal bite cases

Animal bite cases were reported from 12 Upazilas in the Mymensingh district. The highest number of cases was recorded in Mymensingh Sadar Upazila, accounting for 56.5% (8,441 cases) in 2022 and 54.8% (10,419 cases) in 2023. Conversely, the lowest number of cases was reported in

Dhobaura Upazila with 0.70% (105 cases) in 2022 and in Gaffargaon with 0.50% (95 cases) in 2023. Notably, during 2022 and 2023, only four Upazilas (Mymensingh Sadar, Phulpur, Trishal, and Muktagaccha) had a SIR >1. Furthermore, only Mymensingh Sadar Upazila exhibited a SIR >1 in both years **(Table 4).**

Table 4. Spatial distribution of animal bite cases in humans in the Mymensingh district during 2022-2023

Area	Population (2022)	Cases (2022)	Expected cases (2022)	SIR	Population (2023)	Cases (2023)	Expected cases (2023)	SIR
Dhobaura	217466	105 (0.7%)	632	0.17	219467	165 (0.9%)	800	0.21
Fulbaria	495894	727 (4.9%)	1441	0.50	500357	850 (4.5%)	1825	0.47
Phulpur	350967	1518 (10.2%)	1020	**1.49**	355530	508 (2.7%)	1297	0.39
Gaffargaon	463248	130 (0.9%)	1346	0.09	466259	95 (0.5%)	1701	0.06
Gauripur	357331	578 (3.9%)	1038	0.56	360547	679 (3.6%)	1315	0.52
Haluaghat	316528	130 (0.9%)	920	0.14	318997	123 (0.6%)	1163	0.11
Ishwarganj	404598	409 (2.7%)	1176	0.35	407228	541 (2.8%)	1485	0.36
Muktagachha	460381	1146 (7.7%)	1338	0.86	464617	2250 (11.8%)	1695	**1.33**
Nandail	421277	204 (1.4%)	1224	0.17	422962	515 (2.7%)	1543	0.33
Mymensingh Sadar	576927	8441 (56.5%)	1677	**5.03**	591927	10419 (54.8%)	2159	**4.82**
Trishal	491467	1375(9.2%)	1428	0.96	498348	2673 (14.1%)	1818	**1.47**
Bhaluka	583953	180(1.2%)	1697	0.11	600304	180 (0.9%)	2190	0.08
Total	5140037				5206543			
Global Incidence	0.29%				0.36%			
Bangladesh	171,186,372				172,954,319			

3.4. Animal bite risk map

The highest risk of animal bites was observed in 2022 at Mymensingh Sadar Upazila (SIR: 5.03), followed by Phulpur (SIR:1.49). In 2023, the highest risk was also in Mymensingh Sadar (SIR: 4.82), followed by Muktagaccha (SIR: 1.33) and Trishal (SIR: 1.47). The risk map of animal bite cases is presented in **Figure 2(a-b).**

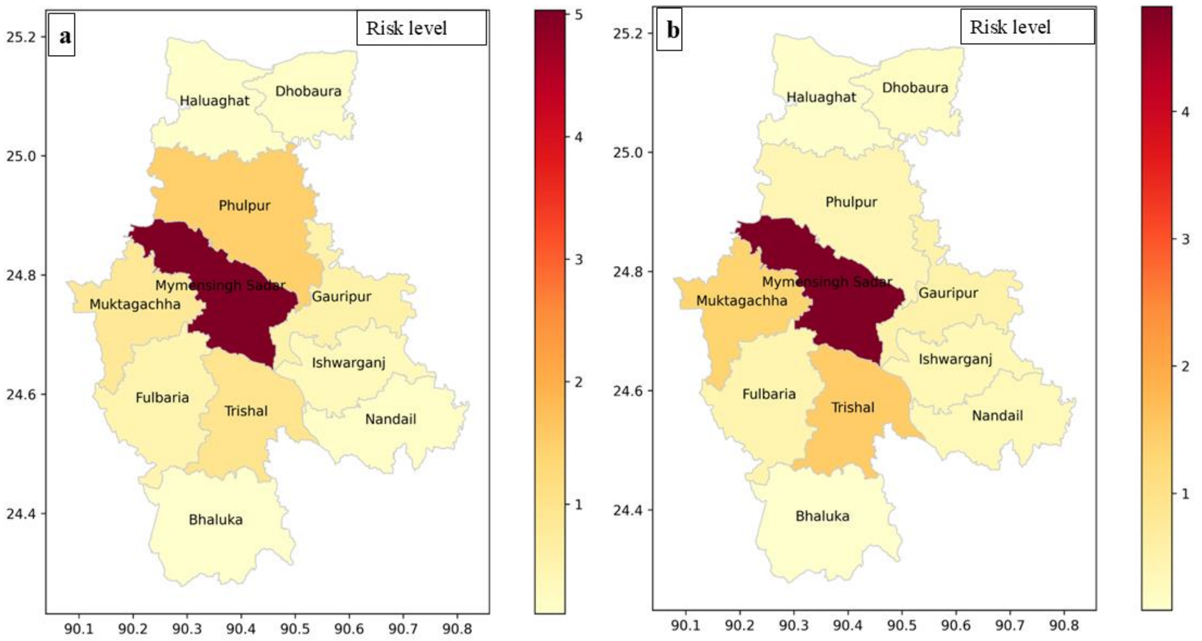

Figure 2(a-b): Animal bite risk map for Mymensingh district in 2022 and 2023. Created in Python using Matplotlib, and Geopandas libraries. Bangladesh Upazila shape files were downloaded from the GADM maps and data (https://gadm.org/).

3.5. Seasonality and annual trend in animal bite cases from 2016 to 2024

The time series analysis indicates both seasonal variation and an increasing trend in animal bite cases **(Figure 3)**.

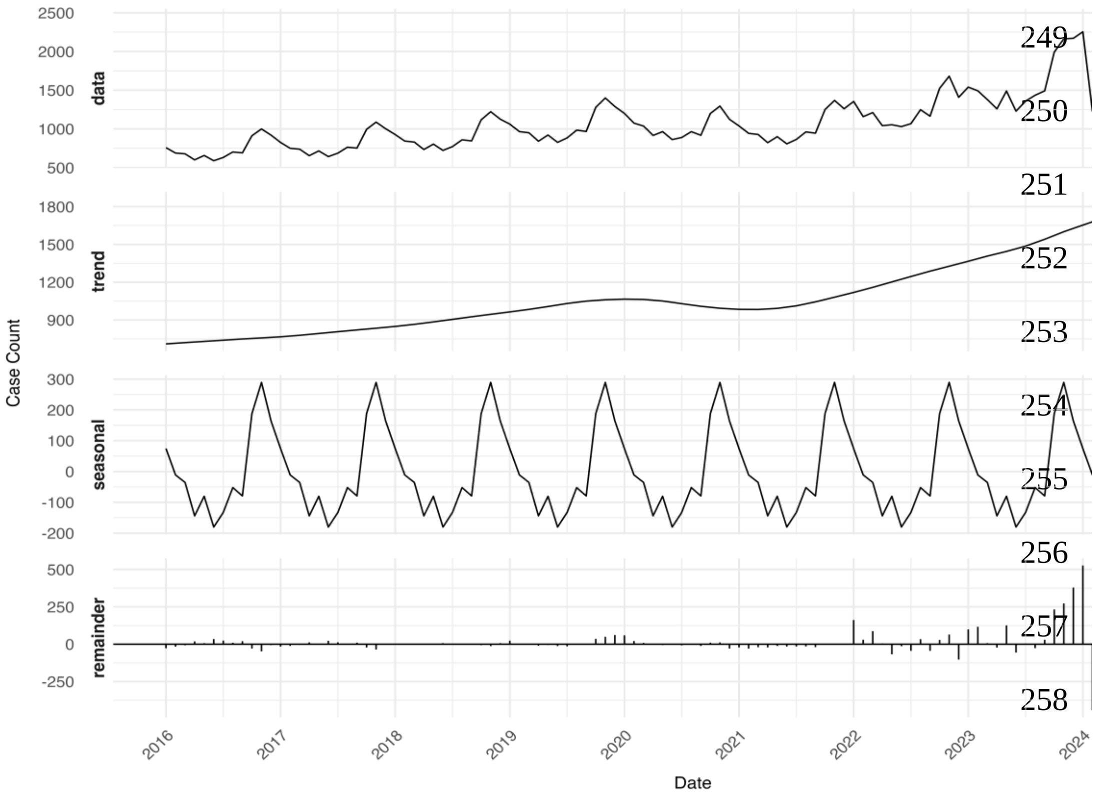

Figure 3: Time series analysis results showing seasonality, trend, and residuals in animal bites cases reported from 2016 to 2024

4. Discussion

Results of this study highlight the need for improvements in healthcare practices and treatment protocols, particularly to ensure timely vaccination delivery. The increasing trend in animal bite cases highlights the need to enhance surveillance and control measures, especially in the veterinary sector. Targeted awareness campaigns for high-risk human groups, such as males and children under 14 years of age, together with preventive programs focused on dogs, are essential to reduce the risk of rabies transmission. Coordinated efforts using a One Health approach among healthcare professionals, veterinarians, policymakers, and community stakeholders are critical for effectively

mitigating the incidence of rabies cases, safeguarding public health, and achieving the goal of eradicating dog-mediated rabies by 2030 in the region.

The annual trends observed in this study reveal a noteworthy increase in the proportion of animal bites within the Mymensingh district over time. The rising proportion of animal bite injuries underscores the ongoing public health challenge of potential rabies exposure. Animal bite incidents in Uganda have been increasing, similar to the trend in Mymensingh, Bangladesh [22]. Studies in Uganda have consistently shown that young people and males are the most frequently bitten, a pattern we also observed. Although dogs are the main biting species in Uganda and Iran [21-23], cat bites were significantly more common in our study. In Mymensingh, vaccinating dogs and using digital tracking to monitor vaccination status could allow for quick risk assessment of bites. This would help prioritize post-exposure prophylaxis for high-risk cases, such as bites from unvaccinated dogs, and optimize resource allocation. The rise in animal bites may be due to a growing human population and pet ownership, improved data recording quality, and increased community awareness of rabies prevention measures.

Given that rabies is a fatal disease, prompt administration of the vaccine after an incident is crucial. However, the majority of bite cases in this study received the vaccine within the first 24 hours after being bitten. This delay in vaccination may be attributed to a lack of awareness among the general population and a failure to recognize the importance of seeking immediate treatment following an animal bite. Post-exposure prophylaxis (PEP) stands as the most critical life-saving intervention to prevent rabies in humans following exposure (24). The average time taken to administer the vaccine after a bite is critical for the effectiveness of post-exposure prophylaxis (20, 25). Thus, efforts to increase public awareness about the urgency of seeking immediate medical attention and

receiving the vaccine promptly after an animal bite are essential to enhance the timely delivery of post-exposure prophylaxis and mitigate the risk of rabies transmission.

Although the proportion of individuals with incomplete vaccination courses decreased from 10.3% in 2022 to 7.8% in 2023, the persistence of incomplete regimens remains a critical public health concern. This issue may stem from limited access to post-exposure rabies vaccines at hospitals in each Upazila and insufficient awareness regarding the necessity of completing the full vaccine schedule. A study conducted in Vietnam highlighted a similar issue, where despite the availability of vaccines, fewer than 50% of patients completed their intramuscular vaccine regimen (26). This emphasizes the need for intensified efforts to educate the public about the importance of completing the entire vaccination course to ensure optimal protection against rabies.

This study revealed that dogs and cats were the predominant animals involved in bites, consistent with numerous findings worldwide. Dogs accounted for 76–94% of reported bite cases in low- and middle-income countries, while cats accounted for 2–50% globally (11). In urban areas of Bangladesh, there is a trend for keeping companion animals, particularly cats. The mass vaccination campaign for dogs in Bangladesh successfully covers about 82% of dogs in each district, significantly reducing the number of dog bite incidents (17). However, this campaign does not include cats, which may be a factor contributing to the observed increase in cat bite cases.

Additionally, the presence of monkeys, foxes, and mongoose in the study indicates a broader spectrum of animals contributing to bites. The consistency in these findings across studies underscores the reliability of the observation that dogs and cats collectively account for approximately 90% of reported animal bites. Rabies elimination requires mass dog vaccination to stop transmission at the source. Post-exposure prophylaxis must be available for all animal bites, including those from cats, jackals, and mongooses. While dogs are responsible for 90% of human

rabies deaths, cat bites account for 6% (15) in Bangladesh, indicating that non-reservoir species can transmit rabies if they become infected through spillover. This strategy can reduce rabies transmission risks and safeguard human and animal populations.

Individuals can identify rabid animals by observing aggressive behavior, multiple bites, and subsequent death. Traditionally, 68-78% of clinically confirmed rabies cases were reported to healers, leading us to believe that human bite cases were likely from suspected rabid animals. Animal bites are used as a proxy for rabid animal bites and predict human rabies risk. All suspect cases are referred to the Infectious Disease Hospital (NRPCC) for management and data recording. Clinical diagnosis is the standard practice due to cultural and societal practices preventing brain tissue sampling and inadequate laboratory facilities. A study of NRPCC records documented 1327 clinically diagnosed human rabies deaths between 2006 and 2018 (15). Notably, there were no rabies deaths during our study period in the Mymensingh DRPCC.

The highest proportion of bite cases occurred in children aged 14 years and younger. Their physical vulnerability, combined with behaviors such as sudden movements, loud noises, or attempts to approach or handle animals without caution, may increase the likelihood of provoking dog and cat attacks. Additionally, children′s limited ability to recognize warning signs from animals makes them more susceptible to bites (27). Moreover, in the home children interact with dogs and cats more commonly. This observation aligns with findings from another study, which noted that children often experience bites on their heads, necks, shoulders, and upper limbs (28). Reports from the World Health Organization (WHO) further highlight that children below the age of 15 are among the most vulnerable populations susceptible to rabies (11).

The present research findings indicate a significant gender discrepancy in animal bites, with males experiencing approximately twice as many bites as females. This observation is consistent with previous studies, which have also noted a higher incidence of animal bites among males compared to females (29, 30).

The lower extremities of the body, particularly the legs, were found to be predominant sites for animal bites. This observation is consistent with another study, which reported that the majority of bites (73.1%) occurred in the lower extremities (7).

The increase in type 3 wounds may be attributed to several factors, including changes in animal behavior due to resource competition, territoriality, and stress from increased human-animal interactions. Rising human population density and the popularity of domestic pets elevate the risk of severe bites by increasing close human-animal contact, particularly in urban environments where animals may feel threatened. Additionally, improved reporting and monitoring systems enhance the detection and accurate classification of severe wounds, contributing to the observed increase (31). HRIG provides immediate protection by supplying neutralizing antibodies. Previous studies have shown that these antibodies play a crucial role in bridging the gap until the individual's immune system can generate vaccine-mediated antibodies (31).

The monthly distribution of animal bite cases in the current study exhibited variation, with the highest number of cases reported from October to January. Earlier studies have reported similar findings of a higher percentage of bite cases occurring during these months, including November and December (32). A similar trend has been observed in domestic ruminants rabies cases in Bangladesh during December, January, and July (6). While rabies cases peak in December and January due to the dog-breeding cycle and its associated rise in aggressive interactions, the increase observed in July suggests other contributing factors. The monsoon season may drive closer human-

animal contact as animals search for food and shelter, increasing the risk of bite-related virus transmission. Additionally, variations in the incubation period may lead to delayed clinical presentations, resulting in cases outside the expected peak months (6, 33).

The highest risk of bite cases was in Mymensingh Sadar, followed by Phulpur. Underreporting in areas might lead to lower proportions. Rabies underreporting often occurs due to poor surveillance and diagnostic challenges, resulting in an underestimated disease burden (13). Depending exclusively on clinical diagnoses undermines the reliability of rabies surveillance systems (7). Therefore, the number of recorded animal bite cases in routine surveillance within the Mymensingh district might represent only a portion of the disease burden. Considering the challenges in diagnosing rabies in developing nations and the lack of precise human rabies trend data, surveillance data on animal bites becomes invaluable. This data offers essential insights to enhance rabies surveillance efforts and improve the allocation of medical and veterinary resources (34).

An effective rabies control strategy should prioritize enhancing collaboration and coordination across sectors using a 'One Health' approach, as recommended during the 2015 global rabies conference. Successful control of human rabies in certain Asian regions has been attributed to robust collaboration between human and animal sectors (35). The government of Bangladesh has initiated various strategies to eliminate rabies, including advocacy, communication, and social mobilization (ACSM), modern treatment for animal bites, mass dog vaccination (MDV), and dog population management (35). The Department of Livestock Services (DLS) is actively involved in mass dog vaccination, population control, and advocacy. Their primary focus is on communication and social mobilization to raise awareness through various programs. The Central Disease Investigation Laboratory, a component of DLS, is dedicated to confirming rabies by

examining brain tissue from deceased animals. However, unlike human rabies surveillance, Bangladesh lacks a comprehensive network for monitoring animal rabies. These activities are predominantly project-based, and the department does not have a sustainable rabies control and prevention program. Despite ongoing efforts, the absence of an integrated animal and human rabies surveillance system impedes effective monitoring of transmission patterns. Furthermore, inadequate coordination between human and animal health sectors hinders the implementation of the "One Health" approach. Additionally, uneven public awareness, particularly in rural areas, contributes to delays in seeking timely medical care after bites. To effectively combat rabies, it is imperative to establish a nationwide animal rabies surveillance system. This should be followed by institutionalizing mass dog vaccination through regular government programs. Furthermore, cross-sectoral collaboration should be enhanced, and community-based education should be expanded to improve awareness, prevention, and prompt healthcare access (36).

Our study is limited by its reliance on hospital-based data collection, which might not fully capture the extent of rabies cases in the community. Hospital-based data primarily reflects individuals who seek medical care after experiencing moderate to severe animal bites, thus potentially underestimating the true prevalence of rabies within the population. Conducting community-based research would offer a more comprehensive understanding of the magnitude of the issue by including individuals who may not seek medical attention for animal bites or who may be treated outside of hospital settings. By surveying households and communities, researchers can gather data on unreported or untreated animal bites, as well as attitudes, beliefs, and practices related to rabies prevention and management.

5. Conclusion

The findings of our study indicate several areas where improvements are needed in healthcare practices and treatment protocols related to rabies prevention and management. One important gap identified is the delay in delivering timely vaccinations following animal bites. This delay poses a considerable risk to individuals exposed to rabies and highlights the need for simplified processes to ensure prompt vaccination delivery. Another gap is animal bites and rabies surveillance in the veterinary sector. The rise in rabies cases highlights the importance of strengthening disease surveillance systems to detect and report cases promptly, as well as implementing targeted interventions to prevent further transmission. To address these challenges, targeted awareness campaigns and preventive measures tailored to high-risk groups are essential. This includes educating the public, particularly males and children under 14 years old who are more susceptible to animal bites, about the importance of seeking immediate medical attention and receiving timely vaccinations after exposure to rabies. Additionally, initiatives aimed at promoting responsible pet ownership practices and increasing vaccination coverage among dogs can help reduce the risk of rabies transmission within communities. Achieving these goals requires coordinated efforts among healthcare professionals, veterinarians, policymakers, community stakeholders and a One Health approach. Collaboration across sectors is crucial for developing and implementing comprehensive rabies control and prevention strategies. By working together, we can effectively mitigate the incidence of rabies cases, safeguard public health, and work towards the goal of eliminating dog-mediated rabies by 2030 in the region.

Acknowledgements

We extend our heartfelt appreciation to SKF Hospital for their invaluable assistance in furnishing the requisite data for the research. Their generosity and gracious support throughout the process are sincerely acknowledged.

Funding: This work was carried out without the support of any specific funding.

Competing interests: The authors have declared that no competing interests exist.

References

1. Dodds WJ, Larson LJ, Christine KL, Schultz RD. Duration of immunity after rabies vaccination in dogs: The Rabies Challenge Fund research study. Canadian Journal of Veterinary Research. 2020;84(2):153-8.
2. Mandal K, Haldar SR, Satapathy DM. Geospatial Distribution and Sociodemographic Profile of Animal Bite Cases attending Anti-rabies Clinic of a Tertiary Care Hospital in Southern Odisha. APCRI Journal. 2022;24(2):1-6.
3. Masthi N, Vairavasolai P. Estimation of animal bites using GPS and google earth in an urban low income area of Bengaluru, South India. APCRI Journal. 2016;17(II):6-10.
4. Manning SE, Rupprecht CE, Fishbein D, Hanlon CA, Lumlertdacha B, Guerra M, et al. Human rabies prevention-United states, 2008. MMWR Recomm Rep. 2008;57:1-28.
5. Amiri S, Maleki Z, Nikbakht H-A, Hassanipour S, Salehiniya H, Ghayour A-R, et al. Epidemiological Patterns of Animal Bites in the Najafabad, Center of Iran (2012–2017). Annals of Global Health. 2020;86(1).
6. Noman Z, Anika T, Haque Z, Rahman A, Ward M, Martínez-López B. Risk factors for rabid animal bites: a study in domestic ruminants in Mymensingh district, Bangladesh. Epidemiology & Infection. 2021;149:e76.
7. Rohde RE, Rupprecht CE. Update on lyssaviruses and rabies: will past progress play as prologue in the near term towards future elimination? Faculty reviews. 2020;9.
8. Pattanayak S, Malla TK, Bara BK, Behera MK. Epidemiological study of animal bite victims and admission in general surgery department, in Southern Odisha: a cross sectional institutional study. International Surgery Journal. 2017;4(10):3470-3.
9. Hossain M, Bulbul T, Ahmed K, Ahmed Z, Salimuzzaman M, Haque MS, et al. Five-year (January 2004–December 2008) surveillance on animal bite and rabies vaccine utilization in the Infectious Disease Hospital, Dhaka, Bangladesh. Vaccine. 2011;29(5):1036-40.
10. Cleaveland S, Hampson K. Rabies elimination research: juxtaposing optimism, pragmatism and realism. Proceedings of the Royal Society B: Biological Sciences. 2017;284(1869):20171880.
11. World Health Organization. Zero by 30: the global strategic plan to end human deaths from dog-mediated rabies by 2030. 2018.
12. Fooks AR, Banyard AC, Horton DL, Johnson N, McElhinney LM, Jackson AC. Current status of rabies and prospects for elimination. The Lancet. 2014;384(9951):1389-99.

13. World Health Organization. WHO expert consultation on rabies: second report: World Health Organization; 2013.
14. Meske M, Fanelli A, Rocha F, Awada L, Soto PC, Mapitse N, et al. Evolution of rabies in South America and inter-species dynamics (2009–2018). Tropical Medicine and Infectious Disease. 2021;6(2):98.
15. Ghosh S, Rana MS, Islam MK, Chowdhury S, Haider N, Kafi MAH, et al. Trends and clinico-epidemiological features of human rabies cases in Bangladesh 2006–2018. Scientific reports. 2020;10(1):2410.
16. Nel LH. Discrepancies in data reporting for rabies, Africa. Emerging infectious diseases. 2013;19(4):529.
17. Ghosh S, Hasan MN, Nath ND, Haider N, Jones DH, Islam MK, Rahaman MM, Mursalin HS, Mahmud N, Kamruzzaman M, Rabby MF. Rabies control in Bangladesh and prediction of human rabies cases by 2030: a One Health approach. The Lancet Regional Health-Southeast Asia. 2024 Aug 1;27.
18. Ghosh S, Chowdhury S, Haider N, Bhowmik RK, Rana MS, Prue Marma AS, et al. Awareness of rabies and response to dog bites in a Bangladesh community. Veterinary medicine and science. 2016;2(3):161-9.
19. Mapatse MF. Public health awareness and seroprevalence of rabies in dogs in Limpopo National Park, and the phylogeny of rabies virus in Mozambique: University of Pretoria; 2021.
20. Liu Q, Wang X, Liu B, Gong Y, Mkandawire N, Li W, et al. Improper wound treatment and delay of rabies post-exposure prophylaxis of animal bite victims in China: Prevalence and determinants. PLoS neglected tropical diseases. 2017;11(7):e0005663.
21. Fèvre EM, Kaboyo R, Persson V, Edelsten M, Coleman P, Cleaveland S. The epidemiology of animal bite injuries in Uganda and projections of the burden of rabies. Tropical medicine & international health. 2005;10(8):790-8.
22. Masiira B, Makumbi I, Matovu JK, Ario AR, Nabukenya I, Kihembo C, et al. Long term trends and spatial distribution of animal bite injuries and deaths due to human rabies infection in Uganda, 2001-2015. PloS one. 2018;13(8):e0198568.
23. Nikbakht H, Heydari H, Malakzadeh Kebria R, Yegane Kasgari M, Mirzad M, Hosseini S. Epidemiological patterns of animal bite injuries in victims under 18 year old in Babol, Iran (2010-14). Journal of Babol University of Medical Sciences. 2015;17(11):67-73.
24. Quiambao BP, Dy-Tioco HZ, Dizon RM, Crisostomo ME, Teuwen DE. Rabies post-exposure prophylaxis with purified equine rabies immunoglobulin: one-year follow-up of patients with laboratory-confirmed category III rabies exposure in the Philippines. Vaccine. 2009;27(51):7162-6.
25. Denis M, Knezevic I, Wilde H, Hemachudha T, Briggs D, Knopf L. An overview of the immunogenicity and effectiveness of current human rabies vaccines administered by intradermal route. Vaccine. 2019;37:A99-A106.
26. Tran CH, Afriyie DO, Pham TN, Otsu S, Urabe M, Dang AD, et al. Rabies post-exposure prophylaxis initiation and adherence among patients in Vietnam, 2014–2016. Vaccine. 2019;37:A54-A63.
27. Patel S, Toppo M, Lodha R. An epidemiological study of animal bite cases in a tertiary care center of Bhopal city: a cross-sectional study. Int J Med Sci Public Health. 2017;6(3):1.
28. Overall KL, Love M. Dog bites to humans—demography, epidemiology, injury, and risk. Journal of the American Veterinary Medical Association. 2001;218(12):1923-34.

29. Gebru G, Romha G, Asefa A, Hadush H, Biedemariam M. Risk factors and spatio-temporal patterns of human rabies exposure in Northwestern Tigray, Ethiopia. Annals of global health. 2019;85(1).
30. Kilic B, Unal B, Semin S, Konakci SK. An important public health problem: rabies suspected bites and post-exposure prophylaxis in a health district in Turkey. International journal of infectious diseases. 2006;10(3):248-54.
31. Haradanhalli RS, Fotedar N, Kumari N, Narayana DA. Safety and clinical efficacy of human rabies immunoglobulin in post exposure prophylaxis for category III animal exposures. Human Vaccines & Immunotherapeutics. 2022;18(5):2081024.
32. Kinge KV, Supe AC. Epidemiology of animal bite cases reported to anti-rabies vaccination OPD at a tertiary-care hospital, Nagpur. Int J Med Sci Public Health. 2016;5(8):1579-82.
33. Guo D, Yin W, Yu H, Thill J-C, Yang W, Chen F, et al. The role of socioeconomic and climatic factors in the spatio-temporal variation of human rabies in China. BMC infectious diseases. 2018;18:1-13.
34. Cleaveland S, Fevre EM, Kaare M, Coleman PG. Estimating human rabies mortality in the United Republic of Tanzania from dog bite injuries. Bulletin of the World health Organization. 2002;80(4):304-10.
35. Byrnes H, Britton A, Bhutia T. Eliminating dog-mediated rabies in Sikkim, India: a 10-year pathway to success for the SARAH program. Frontiers in veterinary science. 2017;4:28.
36. Tenzin, Dhand NK, Rai BD, Changlo, Tenzin S, Tsheten K, et al. Community-based study on knowledge, attitudes and perception of rabies in Gelephu, south-central Bhutan. International health. 2012;4(3):210-9.

Supporting Files

Supporting File 1 (.csv): Time series data of human animal bite cases from 2016 to 2024.

Supporting File 2 (.Rmd): R code to analyze time series data

Figure 1

Click here to access/download;Figure;Figure 1.tif

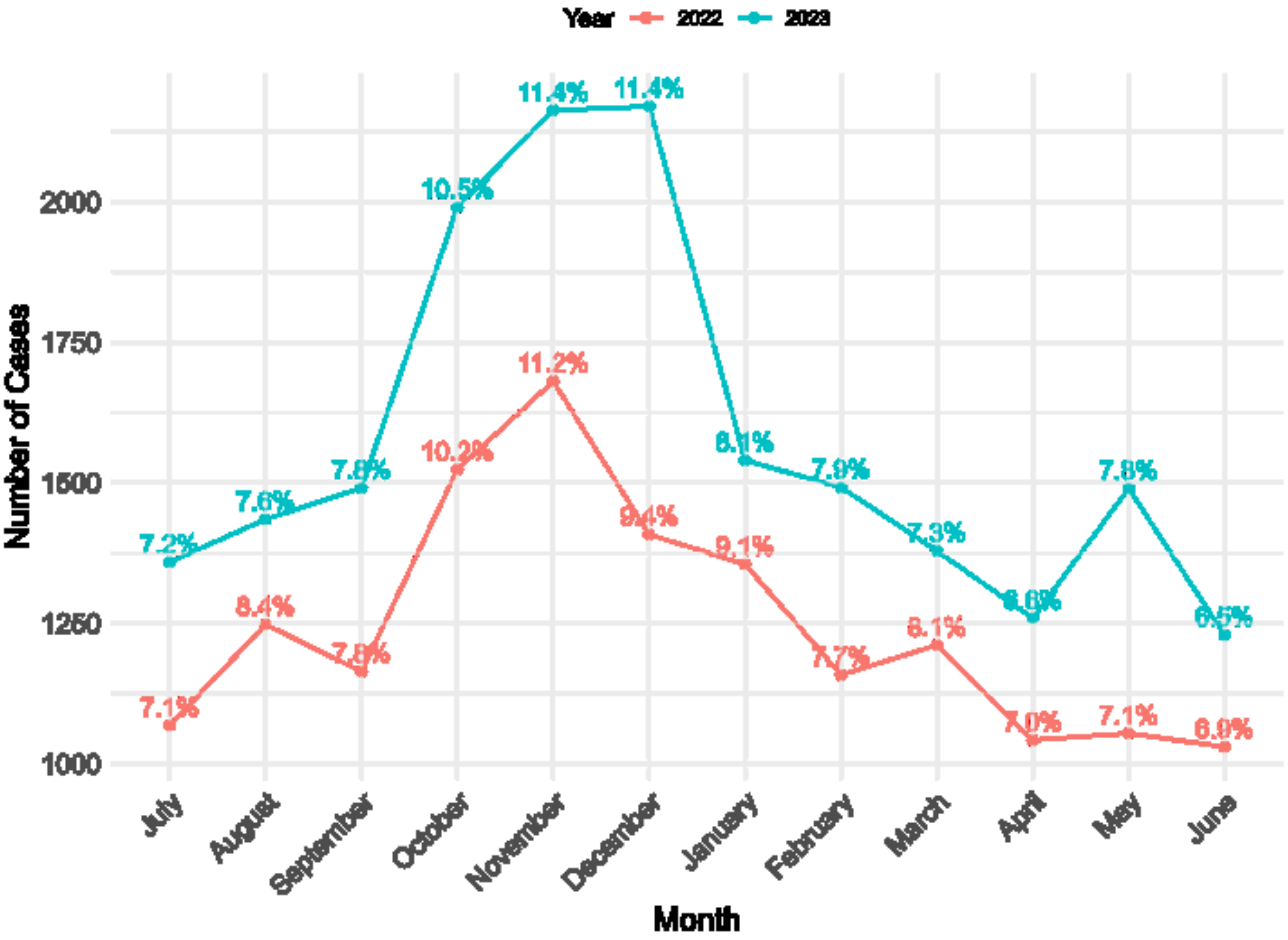

Figure 2

Click here to access/download;Figure;Figure 2.tif

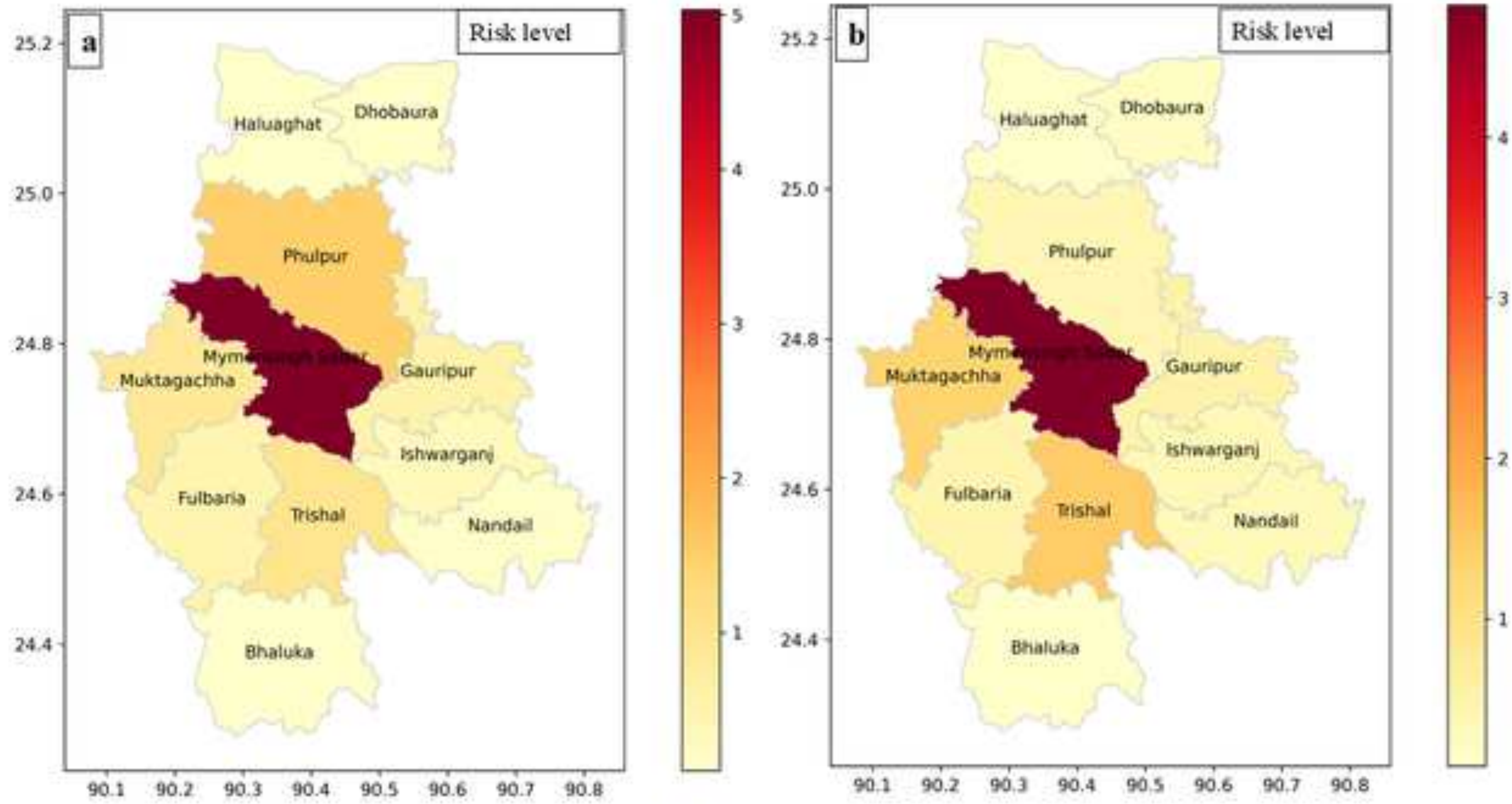

Figure 3

Click here to access/download;Figure;Figure 3.tiff

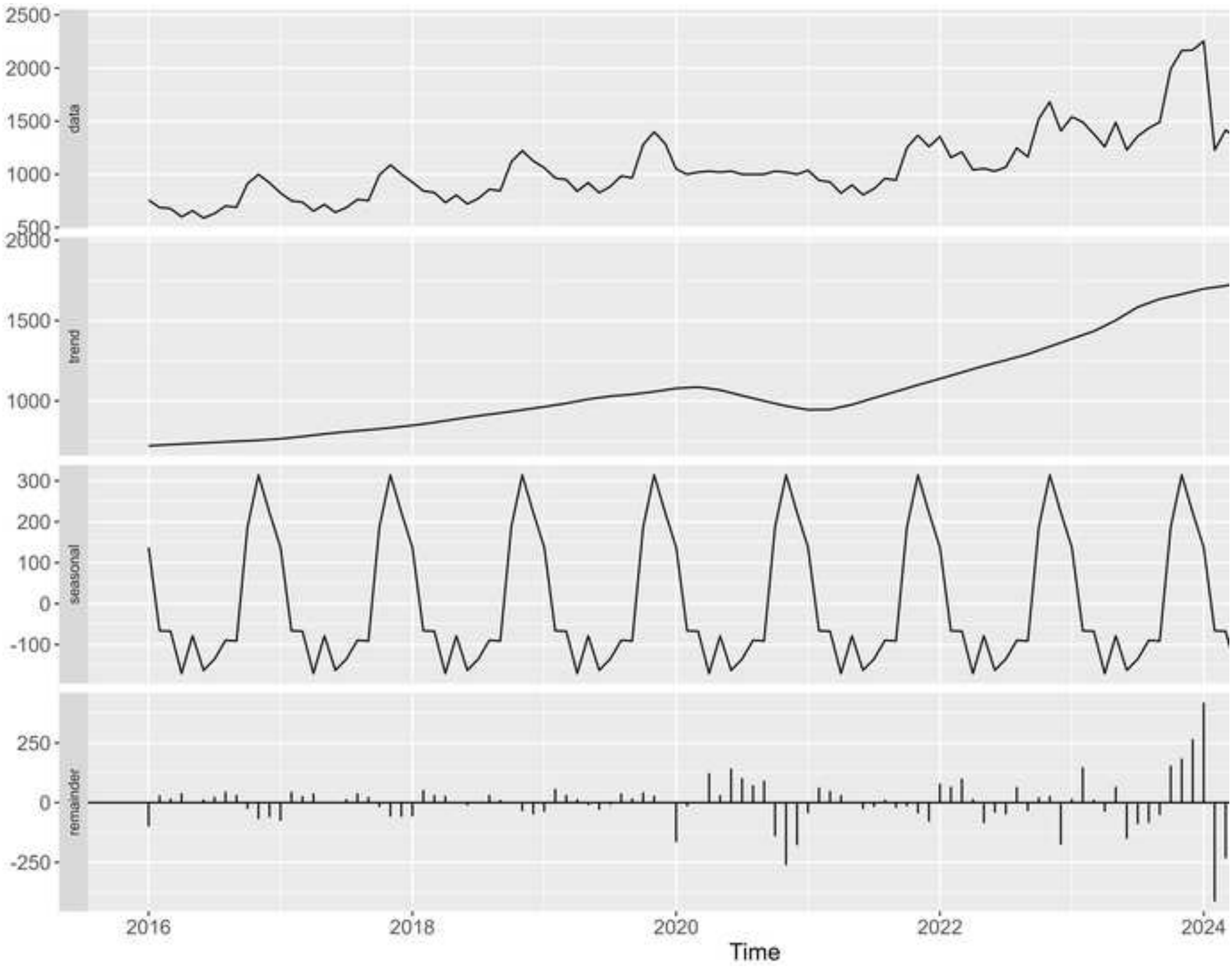

Monthly animal bite cases data

Click here to access/download
Supporting Information
Supplementary _File_1_data_Rabies_16_24.csv

Click here to access/download
Supporting Information
Supplementary_FIle_2_R_code_Animal_bite_time_series
.Rmd

We express our sincere gratitude to both reviewers for their insightful comments, which have significantly enhanced the revised manuscript. All the changes we made based on their feedback are highlighted in blue throughout the revised manuscript.

Reviewer 1

Study title: Demographic, temporal, and spatial analysis of human rabid animal bite cases in Mymensingh District, Bangladesh.

Overall Assessment: The study is a well-designed retrospective analysis, providing valuable insights into the epidemiology of human rabid animal bite cases in Mymensingh District, Bangladesh. The authors have thoroughly examined the demographic, temporal, and spatial characteristics of these cases, offering data that could inform targeted public health interventions. While the findings are robust, certain aspects could be refined. With these improvements, the paper would make a valuable contribution to PLOS Neglected Tropical Diseases

Response: We thank Reviewer #1 for their review and support of publication.

Questions for general understanding/ clarification about the manuscript:

1. Why do we have more cat exposures than dogs exposures? What interventions exists to control cat population in Mymensingh District

Response: This information is provided in the revised manuscript, Lines 304-308.

2. On the abstract, you say that less than 17% of the patients received immediately/promptly, but the following sentence contradicts it by saying that majority received vaccines within the first 24 hours after being bitten, please clarify., also in the results section

Response: We have clarified this matter in both the abstract and results sections.

3. The last sentence of the concluding paragraph of the abstract, do you mean elimination or eradication by 2030?

Response: In the last sentence of the abstract, we have replaced the term “eradication” with “elimination.”

<u>Introduction:</u>

4. Domestic dogs in Bangladesh are generally responsible for about 90% of the reported human rabies cases, and cats contribute to only 6% of the reported human bites cases. What has caused sudden changes in the biting behaviours of cats to supersede domestic dogs in your findings?

Response: In urban areas of Bangladesh, the trend of keeping companion animals, particularly cats, is on the rise. The mass vaccination campaign for dogs in Bangladesh has successfully covered approximately 82% of dogs in each district, significantly reducing the number of dog bite incidents. However, this campaign does not include cats, which may be a contributing factor to the observed increase in the proportion of cat bite cases.

5. Has rabies been eliminated or eradicated in developed countries?. There are very few examples of diseases that have been eradicated but not eliminated globally, rabies being one of them.

Response: We have replaced the term eradicated with eliminated. In developed countries, concerted efforts such as mass vaccination of dogs and administering oral vaccines to wildlife have significantly contributed to the elimination dog-mediated rabies. This has resulted in a subsequent decrease in human rabies cases, although rabies persists in some wildlife populations. We have incorporated this text in the revised manuscript. Lines: 82-85.

6. What kind of misconceptions hinder proper documentation of epidemiological data?, who does the documentation, it's the general public or specific sector, either the health and veterinary sectors? Clarify this

Response: We have clarified the documentation of epidemiological data in lines 90-101.

7. Why are post treatment studies in human bite patients necessary? Does this have do to with the administration of PEP not following the recommended WHO guidelines, are the vaccines not WHO prequalified ?

Response: This text has been removed from the manuscript.

8. What measures are in place in Bangladesh to decrease animal bite incidence? Refer to the last paragraph of your introduction.

Response: We have briefly described the control measures implemented in Bangladesh to mitigate the incidence of animal bites. Lines: 106-111.

<u>Results:</u>

9. How did you differentiate between people bitten by suspicious rabid animals from those bitten by normal health dogs/cats? Is there any post mortem that was conducted on the reported human rabies deaths: no any human rabies deaths reported for the entire period, is that there is none, even due to fatal bites?

Response: Individuals can identify rabid animals by observing their aggressive behavior, multiple bites from a rabid animal and subsequent death of that animal. A substantial majority of clinically confirmed rabies cases (68-78%) were reported to traditional healers. Consequently, we believe that the human bite cases were likely from suspected rabid animals. All rabies suspect cases are referred to the Infectious Disease Hospital (NRPCC) from the District Rabies Prevention and Control Centers (DRPCC) and other areas for management and data recording. Clinical diagnosis is the standard practice due to cultural and societal practices that prevent brain tissue sampling and inadequate laboratory diagnostic facilities in Bangladesh. A study based on NRPCC records documented 1327 clinically diagnosed human rabies deaths between 2006 and 2018 (Reference number 15). Notably, there was no rabies deaths recorded or reported during our study period in the Mymensingh DRPCC.

10. The study does not provide information on the laboratory confirmation of rabies in the reported animal bite cases. This is an important consideration, as clinical diagnoses alone may be unreliable

Response: In response to a previous comment, we have previously stated that laboratory confirmation is not conducted in the reported animal bite cases.

11. The vaccination protocols were revised in 2023 with only three doses given on days 0, 3, and 21, unlike the previous WHO ID recommendations of 4. The presiding sentence states that despite the change, almost all individuals completed the second dose of the vaccine course in both 2022 and 2023. What is the key message here?

Response: We have revised the provided sentence. The three-dose regimen appears to be more effective than the four-dose regimen, as a higher percentage of individuals are completing the prescribed total doses in the revised regimen. Lines: 195-196.

12. What is the key message from the risk map, is this showing the distribution of the high risk cases?

Response: Yes, it is displaying the distribution of high-risk animal bite cases.

13. I don't understand the seasonality of component of the cases reported. Needs more clarification, how this component is related to the observed patterns.

Response: we have provided a more detailed explanation of how seasonality influences the observed patterns in the revised manuscript. Lines: 356-360.

Discussion

14. Yes, from the results its clear that there is an increasing trend in rabid animal bite cases that highlight the need targeted control measures. What kind of surveillance is in place that needs improvement? You only used hospital based data to report on these exposures, where are the veterinary records or how is the veterinary sector involved in this? This would help to highlight the interdisciplinarity of your study

Response: The Department of Livestock Services is involved in mass dog vaccination, population control, and advocacy. They focus on communication and social mobilization to raise awareness through various programs. There is a laboratory dedicated to confirming rabies by examining brain tissue from deceased animals. However, unlike human rabies surveillance, Bangladesh lacks a surveillance network for animal rabies. These activities are project-based, and the department does not have a sustainable rabies control and prevention program. This was discussed in the revised manuscript. Lines: 378-393.

15. In this sentence, ''Targeted awareness campaigns and preventive measures, specifically tailored to high-risk groups such as males, children under 10 years of age, dogs, and cats, are essential", are dogs and cat among the target groups that need awareness?

Response: Thank you for bringing this to our attention. The revised manuscript has been corrected to clarify that dogs and cats are not the target group for awareness. Lines: 43-45; 266-268.

16. In this sentence, you are missing out the key players for rabies elimination, in charge of mass dog vaccination and these are the veterinary sectors "Coordinated efforts among healthcare professionals, policymakers, and community stakeholders are critical for effectively mitigating the incidence of rabies cases, safeguarding public health, and achieving the goal of eradicating dog mediated rabies by 2030 in the region."

Response: Thank you for your feedback. We have incorporated veterinarians into the sentence.

17. From your sentence , that majority of bite exposures received the vaccine within 24 hours, and you state that this delay in vaccination may be attributed to a lack of awareness among the general population, what definition are you using for timely administration of PEP? How soon should this be provided?

Response: By timely administration of PEP, we refer to the immediate administration of the medication following a bite, which is usually done on the same day as the bite.

18. From your findings, cats seem to be contributing more exposures than dogs, contrarily to the findings/reports from other rabies endemic countries. This however is not well captured in your discussion.

Response: The discussion section has addressed this topic.

19. How does the small sized body of a child render him to being prone to attack by dogs and cats? This means any body with a small sized body would be easily attached by a dog/cat?

Response: We have rephrased this text in the revised manuscript. Lines 327-331.

20. Is it called type 3 bites of wound category as per WHO classification of wounds, which is the right terminology to use?

Response: In the revised manuscript, we have replaced type 3 bites with type 3 exposure/wounds.

21. You say that the increase of type 3 wounds could be attributed to changes in animal behaviour, human population density, the popularity of domestic pets, which increase animal contact and risk, and improved reporting and monitoring systems. What causes the change in animal behaviour that may results to someone being attacked by an animal? How is human population density & popularity of domestic pets related to type 3 wounds? Does also improved reporting and monitoring systems have to do with only type 3 wounds?

Response: We have revised the discussion section to address the reviewer's comments and provide clarity. Lines:343-349.

22. You report more cases to be between October and January to be consistent with other findings reporting cases in December, January, and July. Don't you see that July contradicts your statement above, meaning that seasonality is not well captured in your findings. Also, if you can explain or expand of when is the breeding seasons or migration patterns, and during which season of the year do you experience increased animal activity that could be facilitating rabies transmission. This discussion could be strengthened by delving deeper into the potential reasons for the observed seasonal patterns and the implications for targeted interventions

Response: We have refined this discussion in the revised manuscript.

23. This study lacks a One Health approach, particularly in the involvement of veterinarians in disease control. The role of veterinary surveillance is overlooked, which would have strengthened the study's findings and impact.

Response: In the revised manuscript, we have addressed the current status of veterinary surveillance and the role of veterinarians in rabies control.

24. Also detailed description of the current rabies control program, including gaps and planned improvements, would help contextualize the study findings.

Response: We have incorporated additional discussion into the revised manuscript. Lines:378-393.

Reviewer 2

Overview:

The paper explores animal bite cases in Bangladesh in the Mymensingh district. It explores the hospital registers data from 2022 and 2023, as well as the numbers of monthly cases from 2016 to 2023. If the bite incidence is well examined and presented spatially in different upazilas, the link with the rabies burden seems to be lacking. More statistical analysis and data are needed to present a more comprehensive link between animal bite cases and rabies cases. Without this link, there is no way to know the real risk distribution of rabies based on the animal bite cases. The methods should be adapted to include this part.

General remarks:

1. It can be understood that "rabid animal bites" correspond to bites from rabid animals. If so, the rabid status of the animal should have been verified by at least a rapid diagnostic test. Still, it should have been confirmed by an immunofluorescent test or if the animal survived the quarantine period. All this information should be included in the data and the paper. From this work, I have the impression that "rabid animal bite cases" actually correspond to "animal bite cases". If that is the case, I suggest changing the wording to "animal bite cases" in all instances, except for bite cases by confirmed rabid animals. Then, if available, the information on the proportions of such cases should be included in the data.

Response: We have replaced all cases of "rabid animal bites" with "animal bite cases", as suggested by the reviewer.

2. The standard procedure is administering an anti-rabies vaccine for all bite cases, as rabies risk exists. However, you need to explain what is the link between actual rabid cases and animal bite cases. If we consider that all animal bite cases correspond to rabid animal bite cases, then the interventions must probably be more focused on cats than dogs, as you have 52.6% of cat bites. From previous estimations [https://www.ncbi.nlm.nih.gov/pmc/articles/PMC7679256/], cats were responsible for 12.11% of deaths from rabies, whereas dogs were responsible for 81.64%. We can see that the conclusions can be different depending on the point of view. On one hand, we have the probabilities of being bitten; on the other, we must have the probabilities of that animal being rabid to estimate the risk better.

Response: Due to lack of diagnostic facilities at the regional level, it is not possible to differentiate actual rabid cases and animal bite cases. In urban areas of Bangladesh, the trend of keeping companion animals, particularly cats, is on the rise. The mass vaccination campaign for dogs in Bangladesh successfully covers about 82% of dogs in each district, significantly reducing the number of dog bite incidents (Reference number 17). However, this campaign does not include cats, which may be a factor contributing to the observed increase in cat bite cases.

3. Let's also say that in both situations (mentioned in R2), we have a selection bias, so the links between these populations and the general population must be explored. How is the population seeking help characterized compared to the general population (and its counterpart)?b More information must be included on the general populations of the Upazilas and the district. In addition to that, the number of confirmed human rabid cases, the number of known animal rabid cases must be added. This will help to have more robust and generalizable statistical estimates. Otherwise, it can be misleading to use animal bite cases as a surveillance system for rabies.

Response: We appreciate the reviewer's suggestion to explore the relationship between the population seeking help and the general population. In Mymensingh, the overall population distribution is approximately 51% female and 49% male. However, as cultural and behavioral factors influence exposure risk, females are generally less likely to go outside and, consequently, may have a lower likelihood of being bitten by animals. Unfortunately, we do not have age-group-wise population distribution data for Mymensingh, which limits our ability to directly compare the proportion of bite cases with the underlying population structure. Data on the number of confirmed human rabid cases and known animal rabid cases is lacking.

4. If possible, the following information must be included in Table 3:

    - Residence area of the victim: Rural/Urban

Response: Each sub-district encompasses both urban and rural areas. However, specific data on villages or unions were not available to classify cases based on rural and urban regions.

    - Area of the event: Rural/Urban (if the information is unavailable, "Residence" can be used as a proxy)

Response: The same reason explained in the previous comments also renders this impossible.

    - Ownership of the animal: Pet/Stray/Unknown/…

Response: We don't have data on animal ownership.

    - Vaccination status of the animal: Vaccinated/Unvaccinated/…

Response: The vaccination status of the animals was not available.

There is no need to separate the age groups by quartile. The goal is not to have four groups with the same number of persons in those groups but to study the exposure in different age groups. For example, for comparison with other studies, it is better to have the following age groups 0-4, 5-14, 15-24, 25-34, 35-44, 45-54, 55-64, 65+. The 15-24 group can also be divided more, depending on the school system. It can help with providing answers on the relevancy of school awareness programs.

Response: In response to the reviewer's recommendation, we have reorganized the age categories presented in Table 3.

If this information is not collected, it can be part of the recommendation for better surveillance.

5. The following information should be included in Table 4:

- Population of the Upazilas and Demographics information

Response: The population data for each upazila was provided, but their demographic information was not available.

- Number of expected cases in the Upazilas

Response: Now, we have added the expected cases for each upazila in each year in the revised manuscript.

- Total population of the Mymensingh district

Response: The population of Mymensingh District has been added to Table 4.

- Total population of Bangladesh

Response: The total population in Bangladesh is included in table 4.

- Number of cases in the Mymensingh district

Response: We have added the total number of cases in Mymensingh District

- Number of cases in Bangladesh

Response: We don't have data on the number of cases in Bangladesh

- Global incidence in the Mymensingh district

Response: Global incidence in Mymensingh District has been added to Table 4.

- Global incidence in Bangladesh

Response: We lack data on the number of cases in Bangladesh, making it impossible to calculate the global incidence.

- Rural population part of the Upazilas, of the Mymensingh district, and in Bangladesh

Response: We do not have data on the rural population in each Upazila. However, the rural population in Bangladesh has been added.

Some of these elements are important for the SIR calculation and verification.

6. Please provide the monthly data for the animal bite cases and the Jupiter notebook with the time-series analysis. In Figure 3, it seems that the time-series decomposition was based on the yearly data (original time series plot). In that case, the seasonal component makes no sense. The analysis must be redone with monthly data. For Figure 4, what was the intended goal? What is the justification for a Poisson regression instead of a linear one? Why not use the trend from the time-series analysis? The projection of the following years would differ greatly between these models.

Response: We have replaced Figure 4, which previously showed the yearly trend of animal bite cases using Poisson regression analysis. Instead, we now present the time series analysis trend as Figure 3, as suggested by the reviewer. We have revised the analysis using

RStudio instead of Jupyter Notebook. The R-code and monthly data are now available as supplementary files.

Specific remarks

- L. 55 and L. 58. : Please replace “>” with “more than” or similar.

Response: As per the suggestion, the replacement has been made.

- L. 66. : I suggest changing “eradication” to “elimination” (https://www.ncbi.nlm.nih.gov/pmc/articles/PMC2305684/)

Response: The term “eradication” was replaced by “elimination.”

- L. 67. : Please delete “among dogs”; otherwise, there is a contradiction with the first part of the sentence about rabies elimination in the wildlife reservoir.

Response: Deleted as suggested by the reviewer.

- L. 82. : “Notable” is too vague.

Response: We have clarified this statement. Lines 83-86.

- L. 128. : Please change “SIRR” to “SIR” (Standardized Incidence Ratio). The formula is correct, but the usual term is “SIR”.

Response: The term “SIRR” has been corrected to “SIR” as per the reviewer’s suggestion.

- L. 147-148 “human rabid animal bites” See. R1

Response: In the revised manuscript, all instances of human rabid animal bites have been replaced with human animal bites.

- L. 149. : Please change “Promptly” with a more specific term. Ex: “Within 24 hours”.

Response: We have rephrased it as “on the day of the incident”

L. 169 – L. 171. : In my opinion, the distinction between right and left is not that important as this can be a matter of chance. More importantly, legs account for around 55% of all bites, and hands around 34% in 2023.

Response: In the revised manuscript, we have rewritten this section as suggested by the reviewer. Lines: 211-213.

L. 265. – L. 267. “Targeted awareness […] are essential”: Please separate different measures and their target groups. Please, argue what are the preventive measures and how they can be applied to dogs and cats. How would you prioritize it in a resource-constrained setting?

Response: As per the reviewer’s suggestion, we have revised this section. Lines: 266-268.

- L. 272. – L. 275. : Please develop. How do the rising proportions underscore the ongoing public health challenges? How is it aligned with the studies in Uganda and Iran? What parts of these contexts are the same, and what is different? Conducting mass dog vaccinations and tracking this information can help to quickly know the risks of a

particular bite by an owned dog, so it would partially decorrelate the incidence of animal bites and rabies.

Response: We have improved this section of the discussion. Lines: 274-283.

- L. 274. – L. 276. : Please elaborate on different reasons. Is any data on pet ownership or data recording available? If yes, can we see correlations with the animal bite cases? It can at least be done with the human population.

Response: In response to the comment of another reviewer, we have elaborated this.

L. 287. "There was a […] among individuals": I recommend changing this sentence, as "trend" may not reflect the intended meaning. The "trend" would be the decrease in the population proportion with incomplete vaccination (10.3 % in 2022 vs 7.8 % in 2023). However, the number and proportion of people with incomplete vaccination are still issues.

Response: We have changed this sentence in the revised manuscript. Lines: 294-298.

- L. 300. – L. 301. : Please argue why cat vaccination would be as good as mass dog vaccination against rabies. Cat bites represent half of the bites, but is it the same risk? Cats are not a reservoir for rabies, so in the resource-constrained setting, is it really the best choice to target dogs and cats? See R2 and R3.

Response: As per the reviewer's suggestion, we have revised this section. Lines :312-317.

- L. 304. – L. 311. : Please adapt the age groups to align more with other studies.

Response: The age group has been adapted, as also suggested by the other reviewer.

- L. 319. : Is this increase in category 3 bites uniformly distributed over all age levels and genders? Does this increase concern more rural or urban populations? Please add some details if possible.

Response: It varied according age and sex of the cases. We have added this in the results section. Lines: 203-205.

L. 339. – L. 344. : For animal bites to be a reliable surveillance system for rabies, you still need to estimate the rabies burden in each animal species. Controlling for the selection bias and using the aforementioned estimation, you can develop a statistical model for estimating the risks of each species bites as well as risks for different strata of the population. This methodology would help to prioritize and distribute resources more efficiently. Otherwise, you will overestimate the burden, which could be distributed differently than in reality, leading to a less efficient resource allocation.

Response: Our study uses bite incidence as a practical measure for rabies risk in resource-limited settings, although it does not take into account species-specific differences in rabies prevalence. We plan to conduct future research that includes laboratory confirmation of rabies in animals that bite and utilizes stratified risk modeling to more effectively allocate resources. Until this data is available, we recommend mass dog vaccination and universal access to PEP as crucial strategies, in line with global guidelines.

L.424. and L. 428. : You need to specify that WHO is an organization in your reference management software. It seems that it interprets it as a person’s name. In EndNote, for example, you need to put a comma after the organizational name in the Author field.

Response: Corrected according to the reviewer’s comment.

Click here to access/download
Revised Article with Changes Highlighted
Revised Article with Changes HIghlighted.docx
