# Supplementary material for: Demographic, temporal, and spatial analysis of human animal bite cases in Mymensingh District, Bangladesh
Source: PLoS Negl Trop Dis. 2025 Jun 9;19(6):e0012204. doi: 10.1371/journal.pntd.0012204 (PMC12173383; doi:10.1371/journal.pntd.0012204)
Supplement: Attachment — Submitted filename: Review PLOS Neglected Tropical Diseases.docx [file pntd.0012204.s002.docx]

# Review PLOS Neglected Tropical Diseases (PNTD-D-24-00663**)**

## Overview:

The paper explores animal bite cases in Bangladesh in the Mymensingh district. It explores the hospital registers data from 2022 and 2023, as well as the numbers of monthly cases from 2016 to 2023. If the bite incidence is well examined and presented spatially in different upazilas, the link with the rabies burden seems to be lacking. More statistical analysis and data are needed to present a more comprehensive link between animal bite cases and rabies cases. Without this link, there is no way to know the real risk distribution of rabies based on the animal bite cases. The methods should be adapted to include this part.

## General remarks:

R1. It can be understood that “rabid animal bites” correspond to bites from rabid animals. If so, the rabid status of the animal should have been verified by at least a rapid diagnostic test. Still, it should have been confirmed by an immunofluorescent test or if the animal survived the quarantine period. All this information should be included in the data and the paper. From this work, I have the impression that “rabid animal bite cases” actually correspond to “animal bite cases”. If that is the case, I suggest changing the wording to “animal bite cases” in all instances, except for bite cases by confirmed rabid animals. Then, if available, the information on the proportions of such cases should be included in the data.
R2. The standard procedure is administering an anti-rabies vaccine for all bite cases, as rabies risk exists. However, you need to explain what is the link between actual rabid cases and animal bite cases. If we consider that all animal bite cases correspond to rabid animal bite cases, then the interventions must probably be more focused on cats than dogs, as you have 52.6% of cat bites. From previous estimations [https://www.ncbi.nlm.nih.gov/pmc/articles/PMC7679256/], cats were responsible for 12.11% of deaths from rabies, whereas dogs were responsible for 81.64%. We can see that the conclusions can be different depending on the point of view. On one hand, we have the probabilities of being bitten; on the other, we must have the probabilities of that animal being rabid to estimate the risk better.
R3. Let’s also say that in both situations (mentioned in R2), we have a selection bias, so the links between these populations and the general population must be explored. How is the population seeking help characterized compared to the general population (and its counterpart)? More information must be included on the general populations of the Upazilas and the district. In addition to that, the number of confirmed human rabid cases, the number of known animal rabid cases must be added. This will help to have more robust and generalizable statistical estimates. Otherwise, it can be misleading to use animal bite cases as a surveillance system for rabies.
R4. If possible, the following information must be included in Table 3:
    - Residence area of the victim: Rural/Urban
    - Area of the event: Rural/Urban (if the information is unavailable, “Residence” can be used as a proxy)
    - Ownership of the animal: Pet/Stray/Unknown/…
    - Vaccination status of the animal: Vaccinated/Unvaccinated/…
    - There is no need to separate the age groups by quartile. The goal is not to have four groups with the same number of persons in those groups but to study the exposure in different age groups. For example, for comparison with other studies, it is better to have the following age groups 0-4, 5-14, 15-24, 25-34, 35-44, 45-54, 55-64, 65+. The 15-24 group can also be divided more, depending on the school system. It can help with providing answers on the relevancy of school awareness programs.

If this information is not collected, it can be part of the recommendation for better surveillance.

R5. The following information should be included in Table 4:
    - Population of the Upazilas and Demographics information
    - Number of expected cases in the Upazilas
    - Total population of the Mymensingh district
    - Total population of Bangladesh
    - Number of cases in the Mymensingh district
    - Number of cases in Bangladesh
    - Global incidence in the Mymensingh district
    - Global incidence in Bangladesh
    - Rural population part of the Upazilas, of the Mymensingh district, and in Bangladesh

Some of these elements are important for the SIR calculation and verification.

R6. Please provide the monthly data for the animal bite cases and the Jupiter notebook with the time-series analysis. In Figure 3, it seems that the time-series decomposition was based on the yearly data (original time series plot). In that case, the seasonal component makes no sense. The analysis must be redone with monthly data. For Figure 4, what was the intended goal? What is the justification for a Poisson regression instead of a linear one? Why not use the trend from the time-series analysis? The projection of the following years would differ greatly between these models.

## Specific remarks

- L. 55 and L. 58. : Please replace “>” with “more than” or similar.
- L. 66. : I suggest changing “eradication” to “elimination” (<https://www.ncbi.nlm.nih.gov/pmc/articles/PMC2305684/>)
- L. 67. : Please delete “among dogs”; otherwise, there is a contradiction with the first part of the sentence about rabies elimination in the wildlife reservoir.
- L. 82. : “Notable” is too vague.
- L. 128. : Please change “SIRR” to “SIR” (Standardized Incidence Ratio). The formula is correct, but the usual term is “SIR”.
- L. 147-148 “human rabid animal bites” See. R1
- L. 149. : Please change “Promptly” with a more specific term. Ex: “Within 24 hours”.
- L. 169 – L. 171. : In my opinion, the distinction between right and left is not that important as this can be a matter of chance. More importantly, legs account for around 55% of all bites, and hands around 34% in 2023.
- L. 265. – L. 267. “Targeted awareness […] are essential”: Please separate different measures and their target groups. Please, argue what are the preventive measures and how they can be applied to dogs and cats. How would you prioritize it in a resource-constrained setting?
- L. 272. – L. 275. : Please develop. How do the rising proportions underscore the ongoing public health challenges? How is it aligned with the studies in Uganda and Iran? What parts of these contexts are the same, and what is different? Conducting mass dog vaccinations and tracking this information can help to quickly know the risks of a particular bite by an owned dog, so it would partially decorrelate the incidence of animal bites and rabies.
- L. 274. – L. 276. : Please elaborate on different reasons. Is any data on pet ownership or data recording available? If yes, can we see correlations with the animal bite cases? It can at least be done with the human population.
- L. 287. “There was a […] among individuals”: I recommend changing this sentence, as “trend” may not reflect the intended meaning. The “trend” would be the decrease in the population proportion with incomplete vaccination (10.3 % in 2022 vs 7.8 % in 2023). However, the number and proportion of people with incomplete vaccination are still issues.
- L. 300. – L. 301. : Please argue why cat vaccination would be as good as mass dog vaccination against rabies. Cat bites represent half of the bites, but is it the same risk? Cats are not a reservoir for rabies, so in the resource-constrained setting, is it really the best choice to target dogs and cats? See R2 and R3.
- L. 304. – L. 311. : Please adapt the age groups to align more with other studies.
- L. 319. : Is this increase in category 3 bites uniformly distributed over all age levels and genders? Does this increase concern more rural or urban populations? Please add some details if possible.
- L. 339. – L. 344. : For animal bites to be a reliable surveillance system for rabies, you still need to estimate the rabies burden in each animal species. Controlling for the selection bias and using the aforementioned estimation, you can develop a statistical model for estimating the risks of each species bites as well as risks for different strata of the population. This methodology would help to prioritize and distribute resources more efficiently. Otherwise, you will overestimate the burden, which could be distributed differently than in reality, leading to a less efficient resource allocation.
- L.424. and L. 428. : You need to specify that WHO is an organization in your reference management software. It seems that it interprets it as a person’s name. In EndNote, for example, you need to put a comma after the organizational name in the Author field.
