# Supplementary material for: Demographic, temporal, and spatial analysis of human animal bite cases in Mymensingh District, Bangladesh
Source: PLoS Negl Trop Dis. 2025 Jun 9;19(6):e0012204. doi: 10.1371/journal.pntd.0012204 (PMC12173383; doi:10.1371/journal.pntd.0012204)
Supplement: Attachment — Submitted filename: KLushasi-742_Reviewers for PNTD-D-24-00663.docx [file pntd.0012204.s003.docx]

**Manuscript Number: PNTD-D-24-00663**

Reviewer: Dr Kennedy Lushasi

Study title: Demographic, temporal, and spatial analysis of human rabid animal bite cases in Mymensingh District, Bangladesh.

Overall Assessment: The study is a well-designed retrospective analysis, providing valuable insights into the epidemiology of human rabid animal bite cases in Mymensingh District, Bangladesh. The authors have thoroughly examined the demographic, temporal, and spatial characteristics of these cases, offering data that could inform targeted public health interventions. While the findings are robust, certain aspects could be refined. With these improvements, the paper would make a valuable contribution to PLOS Neglected Tropical Diseases

Questions for general understanding/ clarification about the menuscript:

1. Why do we have more can exposures than dogs exposures? What interventions exists to control cat population in Mymensingh District
2. On the abstract, you say that less than 17% of the patients received immediately/promptly, but the following sentence contradicts it by saying that majority received received vaccines within the first 24 hours after being bitten, please clarify., also in the results section
3. The last sentence of the concluding paragraph of the abstract, do you mean elimination or eradication by 2030?

Introduction:

4. Domestic dogs in Bangladesh are generally responsible for about 90% of the reported human rabies cases, and cants contribute to only 6% of the reported human bites cases. What has caused sudden changes in the biting behaviours of cats to supersede domestic dogs in your findings?
5. Has rabies been eliminated or eradicated in developed countries?. There are very few examples of diseases that have been eradicated but not eliminated globally, rabies being one of them.
6. What kind of misconceptions hinder proper documentation of epidemiological data?, who does the documentation, it’s the general public or specific sector, either the health and veterinary sectors? Clarify this
7. Why are post treatment studies in human bite patients necessary? Does this have do to with the administration of PEP not following the recommended WHO guidelines, are the vaccines not WHO prequalified ?
8. What measures are in place in Bangladesh to decrease animal bite incidence? Refer to the last paragraph of your introduction.

Results:

9. How did you differentiate between people bitten by suspicious rabid animals from those bitten by normal health dogs/cats? Is there any post mortem that was conducted on the reported human rabies deaths: no any human rabies deaths reported for the entire period, is that there is none, even due to fatal bites?
10. The study does not provide information on the laboratory confirmation of rabies in the reported animal bite cases. This is an important consideration, as clinical diagnoses alone may be unreliable
11. The vaccination protocols were revised in 2023 with only three doses given on days 0, 3, and 21, unlike the previous WHO ID recommendations of 4. The presiding sentence states that despite the change, almost all individuals completed the second dose of the vaccine course in both 2022 and 2023. What is the key message here?
12. What is the key message from the risk map, is this showing the distribution of the high risk cases?
13. I don’t understand the seasonality of component of the cases reported. Needs more clarification, how this component is related to the observed patterns.

Discussions

14. Yes, from the results its clear that there is an increasing trend in rabid animal bite cases that highlight the need targeted control measures. What kind of surveillance is in place that needs improvement? You only used hospital based data to report on these exposures, where are the veterinary records or how is the veterinary sector involved in this? This would help to highlight the interdisciplinarity of your study
15. In this sentence, ‘’Targeted awareness campaigns and preventive measures, specifically tailored to high-risk groups such as males, children under 10 years of age, dogs, and cats, are essential”, are dogs and cat among the target groups that need awareness?
16. In this sentence, you are missing out the key players for rabies elimination, in charge of mass dog vaccination and these are the veterinary sectors “Coordinated efforts among healthcare professionals, policymakers, and community stakeholders are critical for effectively mitigating the incidence of rabies cases, safeguarding public health, and achieving 269 the goal of eradicating dog270 mediated rabies by 2030 in the region.”
17. From your sentence , that majority of bite exposures received the vaccine within 24 hours, and you state that this delay in vaccination may be attributed to a lack of awareness among the general population, what definition are you using for timely administration of PEP? How soon should this be provided?
18. From your findings, cats seem to be contributing more exposures than dogs, contrarily to the findings/reports from other rabies endemic countries. This however is not well captured in your discussion
19. How does the small sized body of a child render him to being prone to attack by dogs and cats? This means any body with a small sized body would be easily attached by a dog/cat?
20. Is it called type 3 bites of wound category as per WHO classification of wounds, which is the right terminology to use?
21. You say that the increase of type 3 wounds could be attributed to changes in animal behaviour, human population density, the popularity of domestic pets, which increase animal contact and risk, and improved reporting and monitoring systems. What causes the change in animal behaviour that may results to someone being attacked by an animal? How is human population density & popularity of domestic pets related to type 3 wounds? Does also improved reporting and monitoring systems have to do with only type 3 wounds?
22. You report more cases to be between October and January to be consistent with other findings reporting cases in December, January, and July. Don’t you see that July contradicts your statement above, meaning that seasonality is not well captured in your findings. Also, if you can explain or expand of when is the breeding seasons or migration patterns, and during which season of the year do you experience increased animal activity that could be facilitating rabies transmission. This discussion could be strengthened by delving deeper into the potential reasons for the observed seasonal patterns and the implications for targeted interventions
23. This study lacks a One Health approach, particularly in the involvement of veterinarians in disease control. The role of veterinary surveillance is overlooked, which would have strengthened the study's findings and impact.
24. Also detailed description of the current rabies control program, including gaps and planned improvements, would help contextualize the study findings.
