# Supplementary material for: Demographic, temporal, and spatial analysis of human animal bite cases in Mymensingh District, Bangladesh
Source: PLoS Negl Trop Dis. 2025 Jun 9;19(6):e0012204. doi: 10.1371/journal.pntd.0012204 (PMC12173383; doi:10.1371/journal.pntd.0012204)
Supplement: Attachment — Submitted filename: Response_reviewers_comments.docx [file pntd.0012204.s004.docx]

We express our sincere gratitude to both reviewers for their insightful comments, which have significantly enhanced the revised manuscript. All the changes we made based on their feedback are highlighted in blue throughout the revised manuscript.

**Reviewer 1**

Study title: Demographic, temporal, and spatial analysis of human rabid animal bite cases in Mymensingh District, Bangladesh.

Overall Assessment: The study is a well-designed retrospective analysis, providing valuable insights into the epidemiology of human rabid animal bite cases in Mymensingh District, Bangladesh. The authors have thoroughly examined the demographic, temporal, and spatial characteristics of these cases, offering data that could inform targeted public health interventions. While the findings are robust, certain aspects could be refined. With these improvements, the paper would make a valuable contribution to PLOS Neglected Tropical Diseases

Response: We thank Reviewer #1 for their review and support of publication.

Questions for general understanding/ clarification about the manuscript:

1. Why do we have more cat exposures than dogs exposures? What interventions exists to control cat population in Mymensingh District

Response: This information is provided in the revised manuscript, Lines 304-308.

2. On the abstract, you say that less than 17% of the patients received immediately/promptly, but the following sentence contradicts it by saying that majority received vaccines within the first 24 hours after being bitten, please clarify., also in the results section

Response: We have clarified this matter in both the abstract and results sections.

3. The last sentence of the concluding paragraph of the abstract, do you mean elimination or eradication by 2030?

Response: In the last sentence of the abstract, we have replaced the term “eradication” with “elimination.”

Introduction:

4. Domestic dogs in Bangladesh are generally responsible for about 90% of the reported human rabies cases, and cats contribute to only 6% of the reported human bites cases. What has caused sudden changes in the biting behaviours of cats to supersede domestic dogs in your findings?

Response: In urban areas of Bangladesh, the trend of keeping companion animals, particularly cats, is on the rise. The mass vaccination campaign for dogs in Bangladesh has successfully covered approximately 82% of dogs in each district, significantly reducing the number of dog bite incidents. However, this campaign does not include cats, which may be a contributing factor to the observed increase in the proportion of cat bite cases.

5. Has rabies been eliminated or eradicated in developed countries?. There are very few examples of diseases that have been eradicated but not eliminated globally, rabies being one of them.

Response: We have replaced the term eradicated with eliminated. In developed countries, concerted efforts such as mass vaccination of dogs and administering oral vaccines to wildlife have significantly contributed to the elimination dog-mediated rabies. This has resulted in a subsequent decrease in human rabies cases, although rabies persists in some wildlife populations. We have incorporated this text in the revised manuscript. Lines: 82-85.

6. What kind of misconceptions hinder proper documentation of epidemiological data?, who does the documentation, it’s the general public or specific sector, either the health and veterinary sectors? Clarify this

Response: We have clarified the documentation of epidemiological data in lines 90-101.

7. Why are post treatment studies in human bite patients necessary? Does this have do to with the administration of PEP not following the recommended WHO guidelines, are the vaccines not WHO prequalified ?

Response: This text has been removed from the manuscript.

8. What measures are in place in Bangladesh to decrease animal bite incidence? Refer to the last paragraph of your introduction.

Response: We have briefly described the control measures implemented in Bangladesh to mitigate the incidence of animal bites. Lines: 106-111.

Results:

9. How did you differentiate between people bitten by suspicious rabid animals from those bitten by normal health dogs/cats? Is there any post mortem that was conducted on the reported human rabies deaths: no any human rabies deaths reported for the entire period, is that there is none, even due to fatal bites?

Response: Individuals can identify rabid animals by observing their aggressive behavior, multiple bites from a rabid animal and subsequent death of that animal. A substantial majority of clinically confirmed rabies cases (68-78%) were reported to traditional healers. Consequently, we believe that the human bite cases were likely from suspected rabid animals. All rabies suspect cases are referred to the Infectious Disease Hospital (NRPCC) from the District Rabies Prevention and Control Centers (DRPCC) and other areas for management and data recording. Clinical diagnosis is the standard practice due to cultural and societal practices that prevent brain tissue sampling and inadequate laboratory diagnostic facilities in Bangladesh. A study based on NRPCC records documented 1327 clinically diagnosed human rabies deaths between 2006 and 2018 (Reference number 15). Notably, there was no rabies deaths recorded or reported during our study period in the Mymensingh DRPCC.

10. The study does not provide information on the laboratory confirmation of rabies in the reported animal bite cases. This is an important consideration, as clinical diagnoses alone may be unreliable

Response: In response to a previous comment, we have previously stated that laboratory confirmation is not conducted in the reported animal bite cases.

11. The vaccination protocols were revised in 2023 with only three doses given on days 0, 3, and 21, unlike the previous WHO ID recommendations of 4. The presiding sentence states that despite the change, almost all individuals completed the second dose of the vaccine course in both 2022 and 2023. What is the key message here?

Response: We have revised the provided sentence. The three-dose regimen appears to be more effective than the four-dose regimen, as a higher percentage of individuals are completing the prescribed total doses in the revised regimen. Lines: 195-196.

12. What is the key message from the risk map, is this showing the distribution of the high risk cases?

Response: Yes, it is displaying the distribution of high-risk animal bite cases.

13. I don’t understand the seasonality of component of the cases reported. Needs more clarification, how this component is related to the observed patterns.

Response: we have provided a more detailed explanation of how seasonality influences the observed patterns in the revised manuscript. Lines: 356-360.

Discussion

14. Yes, from the results its clear that there is an increasing trend in rabid animal bite cases that highlight the need targeted control measures. What kind of surveillance is in place that needs improvement? You only used hospital based data to report on these exposures, where are the veterinary records or how is the veterinary sector involved in this? This would help to highlight the interdisciplinarity of your study

Response: The Department of Livestock Services is involved in mass dog vaccination, population control, and advocacy. They focus on communication and social mobilization to raise awareness through various programs. There is a laboratory dedicated to confirming rabies by examining brain tissue from deceased animals. However, unlike human rabies surveillance, Bangladesh lacks a surveillance network for animal rabies. These activities are project-based, and the department does not have a sustainable rabies control and prevention program. This was discussed in the revised manuscript. Lines: 378-393.

15. In this sentence, ‘’Targeted awareness campaigns and preventive measures, specifically tailored to high-risk groups such as males, children under 10 years of age, dogs, and cats, are essential”, are dogs and cat among the target groups that need awareness?

Response: Thank you for bringing this to our attention. The revised manuscript has been corrected to clarify that dogs and cats are not the target group for awareness. Lines: 43-45; 266-268.

16. In this sentence, you are missing out the key players for rabies elimination, in charge of mass dog vaccination and these are the veterinary sectors “Coordinated efforts among healthcare professionals, policymakers, and community stakeholders are critical for effectively mitigating the incidence of rabies cases, safeguarding public health, and achieving the goal of eradicating dog mediated rabies by 2030 in the region.”

Response: Thank you for your feedback. We have incorporated veterinarians into the sentence.

17. From your sentence , that majority of bite exposures received the vaccine within 24 hours, and you state that this delay in vaccination may be attributed to a lack of awareness among the general population, what definition are you using for timely administration of PEP? How soon should this be provided?

Response: By timely administration of PEP, we refer to the immediate administration of the medication following a bite, which is usually done on the same day as the bite.

18. From your findings, cats seem to be contributing more exposures than dogs, contrarily to the findings/reports from other rabies endemic countries. This however is not well captured in your discussion.

Response: The discussion section has addressed this topic.

19. How does the small sized body of a child render him to being prone to attack by dogs and cats? This means any body with a small sized body would be easily attached by a dog/cat?

Response: We have rephrased this text in the revised manuscript. Lines 327-331.

20. Is it called type 3 bites of wound category as per WHO classification of wounds, which is the right terminology to use?

Response: In the revised manuscript, we have replaced type 3 bites with type 3 exposure/wounds.

21. You say that the increase of type 3 wounds could be attributed to changes in animal behaviour, human population density, the popularity of domestic pets, which increase animal contact and risk, and improved reporting and monitoring systems. What causes the change in animal behaviour that may results to someone being attacked by an animal? How is human population density & popularity of domestic pets related to type 3 wounds? Does also improved reporting and monitoring systems have to do with only type 3 wounds?

Response: We have revised the discussion section to address the reviewer’s comments and provide clarity. Lines:343-349.

22. You report more cases to be between October and January to be consistent with other findings reporting cases in December, January, and July. Don’t you see that July contradicts your statement above, meaning that seasonality is not well captured in your findings. Also, if you can explain or expand of when is the breeding seasons or migration patterns, and during which season of the year do you experience increased animal activity that could be facilitating rabies transmission. This discussion could be strengthened by delving deeper into the potential reasons for the observed seasonal patterns and the implications for targeted interventions

Response: We have refined this discussion in the revised manuscript.

23. This study lacks a One Health approach, particularly in the involvement of veterinarians in disease control. The role of veterinary surveillance is overlooked, which would have strengthened the study's findings and impact.

Response: In the revised manuscript, we have addressed the current status of veterinary surveillance and the role of veterinarians in rabies control.

24. Also detailed description of the current rabies control program, including gaps and planned improvements, would help contextualize the study findings.

Response: We have incorporated additional discussion into the revised manuscript. Lines:378-393.

Reviewer 2

## Overview:

The paper explores animal bite cases in Bangladesh in the Mymensingh district. It explores the hospital registers data from 2022 and 2023, as well as the numbers of monthly cases from 2016 to 2023. If the bite incidence is well examined and presented spatially in different upazilas, the link with the rabies burden seems to be lacking. More statistical analysis and data are needed to present a more comprehensive link between animal bite cases and rabies cases. Without this link, there is no way to know the real risk distribution of rabies based on the animal bite cases. The methods should be adapted to include this part.

## General remarks:

1. It can be understood that “rabid animal bites” correspond to bites from rabid animals. If so, the rabid status of the animal should have been verified by at least a rapid diagnostic test. Still, it should have been confirmed by an immunofluorescent test or if the animal survived the quarantine period. All this information should be included in the data and the paper. From this work, I have the impression that “rabid animal bite cases” actually correspond to “animal bite cases”. If that is the case, I suggest changing the wording to “animal bite cases” in all instances, except for bite cases by confirmed rabid animals. Then, if available, the information on the proportions of such cases should be included in the data.

Response: We have replaced all cases of “rabid animal bites” with “animal bite cases”, as suggested by the reviewer.

2. The standard procedure is administering an anti-rabies vaccine for all bite cases, as rabies risk exists. However, you need to explain what is the link between actual rabid cases and animal bite cases. If we consider that all animal bite cases correspond to rabid animal bite cases, then the interventions must probably be more focused on cats than dogs, as you have 52.6% of cat bites. From previous estimations [https://www.ncbi.nlm.nih.gov/pmc/articles/PMC7679256/], cats were responsible for 12.11% of deaths from rabies, whereas dogs were responsible for 81.64%. We can see that the conclusions can be different depending on the point of view. On one hand, we have the probabilities of being bitten; on the other, we must have the probabilities of that animal being rabid to estimate the risk better.

Response: Due to lack of diagnostic facilities at the regional level, it is not possible to differentiate actual rabid cases and animal bite cases. In urban areas of Bangladesh, the trend of keeping companion animals, particularly cats, is on the rise. The mass vaccination campaign for dogs in Bangladesh successfully covers about 82% of dogs in each district, significantly reducing the number of dog bite incidents (Reference number 17). However, this campaign does not include cats, which may be a factor contributing to the observed increase in cat bite cases.

3. Let’s also say that in both situations (mentioned in R2), we have a selection bias, so the links between these populations and the general population must be explored. How is the population seeking help characterized compared to the general population (and its counterpart)?b More information must be included on the general populations of the Upazilas and the district. In addition to that, the number of confirmed human rabid cases, the number of known animal rabid cases must be added. This will help to have more robust and generalizable statistical estimates. Otherwise, it can be misleading to use animal bite cases as a surveillance system for rabies.

Response: We appreciate the reviewer’s suggestion to explore the relationship between the population seeking help and the general population. In Mymensingh, the overall population distribution is approximately 51% female and 49% male. However, as cultural and behavioral factors influence exposure risk, females are generally less likely to go outside and, consequently, may have a lower likelihood of being bitten by animals. Unfortunately, we do not have age-group-wise population distribution data for Mymensingh, which limits our ability to directly compare the proportion of bite cases with the underlying population structure. Data on the number of confirmed human rabid cases and known animal rabid cases is lacking.

4. If possible, the following information must be included in Table 3:
   - Residence area of the victim: Rural/Urban

Response: Each sub-district encompasses both urban and rural areas. However, specific data on villages or unions were not available to classify cases based on rural and urban regions.

- Area of the event: Rural/Urban (if the information is unavailable, “Residence” can be used as a proxy)

Response: The same reason explained in the previous comments also renders this impossible.

- Ownership of the animal: Pet/Stray/Unknown/…

Response: We don’t have data on animal ownership.

- Vaccination status of the animal: Vaccinated/Unvaccinated/…

Response: The vaccination status of the animals was not available.

There is no need to separate the age groups by quartile. The goal is not to have four groups with the same number of persons in those groups but to study the exposure in different age groups. For example, for comparison with other studies, it is better to have the following age groups 0-4, 5-14, 15-24, 25-34, 35-44, 45-54, 55-64, 65+. The 15-24 group can also be divided more, depending on the school system. It can help with providing answers on the relevancy of school awareness programs.

Response: In response to the reviewer’s recommendation, we have reorganized the age categories presented in Table 3.

If this information is not collected, it can be part of the recommendation for better surveillance.

5. The following information should be included in Table 4:
   - Population of the Upazilas and Demographics information

Response: The population data for each upazila was provided, but their demographic information was not available.

- Number of expected cases in the Upazilas

Response: Now, we have added the expected cases for each upazila in each year in the revised manuscript.

- Total population of the Mymensingh district

Response: The population of Mymensingh District has been added to Table 4.

- Total population of Bangladesh

Response: The total population in Bangladesh is included in table 4.

- Number of cases in the Mymensingh district

Response: We have added the total number of cases in Mymensingh District

- Number of cases in Bangladesh

Response: We don’t have data on the number of cases in Bangladesh

- Global incidence in the Mymensingh district

Response: Global incidence in Mymensingh District has been added to Table 4.

- Global incidence in Bangladesh

Response: We lack data on the number of cases in Bangladesh, making it impossible to calculate the global incidence.

- Rural population part of the Upazilas, of the Mymensingh district, and in Bangladesh

Response: We do not have data on the rural population in each Upazila. However, the rural population in Bangladesh has been added.

Some of these elements are important for the SIR calculation and verification.

6. Please provide the monthly data for the animal bite cases and the Jupiter notebook with the time-series analysis. In Figure 3, it seems that the time-series decomposition was based on the yearly data (original time series plot). In that case, the seasonal component makes no sense. The analysis must be redone with monthly data. For Figure 4, what was the intended goal? What is the justification for a Poisson regression instead of a linear one? Why not use the trend from the time-series analysis? The projection of the following years would differ greatly between these models.

Response: We have replaced Figure 4, which previously showed the yearly trend of animal bite cases using Poisson regression analysis. Instead, we now present the time series analysis trend as Figure 3, as suggested by the reviewer. We have revised the analysis using RStudio instead of Jupyter Notebook. The R-code and monthly data are now available as supplementary files.

Specific remarks

- L. 55 and L. 58. : Please replace “>” with “more than” or similar.

Response: As per the suggestion, the replacement has been made.

- L. 66. : I suggest changing “eradication” to “elimination” (<https://www.ncbi.nlm.nih.gov/pmc/articles/PMC2305684/>)

Response: The term “eradication” was replaced by “elimination.”

- L. 67. : Please delete “among dogs”; otherwise, there is a contradiction with the first part of the sentence about rabies elimination in the wildlife reservoir.

Response: Deleted as suggested by the reviewer.

- L. 82. : “Notable” is too vague.

Response: We have clarified this statement. Lines 83-86.

- L. 128. : Please change “SIRR” to “SIR” (Standardized Incidence Ratio). The formula is correct, but the usual term is “SIR”.

Response: The term “SIRR” has been corrected to “SIR” as per the reviewer’s suggestion.

- L. 147-148 “human rabid animal bites” See. R1

Response: In the revised manuscript, all instances of human rabid animal bites have been replaced with human animal bites.

- L. 149. : Please change “Promptly” with a more specific term. Ex: “Within 24 hours”.

Response: We have rephrased it as “on the day of the incident”

L. 169 – L. 171. : In my opinion, the distinction between right and left is not that important as this can be a matter of chance. More importantly, legs account for around 55% of all bites, and hands around 34% in 2023.

Response: In the revised manuscript, we have rewritten this section as suggested by the reviewer. Lines: 211-213.

L. 265. – L. 267. “Targeted awareness […] are essential”: Please separate different measures and their target groups. Please, argue what are the preventive measures and how they can be applied to dogs and cats. How would you prioritize it in a resource-constrained setting?

Response: As per the reviewer’s suggestion, we have revised this section. Lines: 266-268.

- L. 272. – L. 275. : Please develop. How do the rising proportions underscore the ongoing public health challenges? How is it aligned with the studies in Uganda and Iran? What parts of these contexts are the same, and what is different? Conducting mass dog vaccinations and tracking this information can help to quickly know the risks of a particular bite by an owned dog, so it would partially decorrelate the incidence of animal bites and rabies.

Response: We have improved this section of the discussion. Lines: 274-283.

- L. 274. – L. 276. : Please elaborate on different reasons. Is any data on pet ownership or data recording available? If yes, can we see correlations with the animal bite cases? It can at least be done with the human population.

Response: In response to the comment of another reviewer, we have elaborated this.

L. 287. “There was a […] among individuals”: I recommend changing this sentence, as “trend” may not reflect the intended meaning. The “trend” would be the decrease in the population proportion with incomplete vaccination (10.3 % in 2022 vs 7.8 % in 2023). However, the number and proportion of people with incomplete vaccination are still issues.

Response: We have changed this sentence in the revised manuscript. Lines: 294-298.

- L. 300. – L. 301. : Please argue why cat vaccination would be as good as mass dog vaccination against rabies. Cat bites represent half of the bites, but is it the same risk? Cats are not a reservoir for rabies, so in the resource-constrained setting, is it really the best choice to target dogs and cats? See R2 and R3.

Response: As per the reviewer’s suggestion, we have revised this section. Lines :312-317.

- L. 304. – L. 311. : Please adapt the age groups to align more with other studies.

Response: The age group has been adapted, as also suggested by the other reviewer.

- L. 319. : Is this increase in category 3 bites uniformly distributed over all age levels and genders? Does this increase concern more rural or urban populations? Please add some details if possible.

Response: It varied according age and sex of the cases. We have added this in the results section. Lines: 203-205.

L. 339. – L. 344. : For animal bites to be a reliable surveillance system for rabies, you still need to estimate the rabies burden in each animal species. Controlling for the selection bias and using the aforementioned estimation, you can develop a statistical model for estimating the risks of each species bites as well as risks for different strata of the population. This methodology would help to prioritize and distribute resources more efficiently. Otherwise, you will overestimate the burden, which could be distributed differently than in reality, leading to a less efficient resource allocation.

Response: Our study uses bite incidence as a practical measure for rabies risk in resource-limited settings, although it does not take into account species-specific differences in rabies prevalence. We plan to conduct future research that includes laboratory confirmation of rabies in animals that bite and utilizes stratified risk modeling to more effectively allocate resources. Until this data is available, we recommend mass dog vaccination and universal access to PEP as crucial strategies, in line with global guidelines.

L.424. and L. 428. : You need to specify that WHO is an organization in your reference management software. It seems that it interprets it as a person’s name. In EndNote, for example, you need to put a comma after the organizational name in the Author field.

Response: Corrected according to the reviewer’s comment.
