# Supplementary material for: Demographic, temporal, and spatial analysis of human animal bite cases in Mymensingh District, Bangladesh
Source: PLoS Negl Trop Dis. 2025 Jun 9;19(6):e0012204. doi: 10.1371/journal.pntd.0012204 (PMC12173383; doi:10.1371/journal.pntd.0012204)
Supplement: Attachment — Submitted filename: Response to Reviewers.docx [file pntd.0012204.s006.docx]

Response to reviewers’ comments

Reviewer #2: The manuscript is suitable for publication pending the following very minor changes:

Minor Suggestion: Mention if any ethics approval was obtained, even for retrospective hospital data, for completeness.

Response: This study used anonymized, retrospective hospital records and aggregated public health data. As this was a secondary data analysis, ethical approval was not necessary. This information has now been included in the method section of the revised manuscript.

1. Clarify the perceived contradiction between "majority received PEP within 24 hours" and the "significant gap in timely vaccination.

Response: We have clarified this in the revised manuscript. Lines: 31-33, 38-39.

2. Mention or hypothesize why older age groups (55–64) had more type 3 bites.

Response: We have outlined the likely causes of type 3 bites in older individuals. Lines: 357-364.

3. Add a sentence or paragraph highlighting the strengths of the study.

Response: We have added our study’s strength before discussing limitations. Lines: 410-416.

4. Slightly improve Figure 3 visual quality.

Response: The resolution and font size of the text in Figure 3 have been increased.

Reviewer #3:

Are data incomplete or not available for 2020?

Response: Data for 2020 were not available.

The link between the bite and rabies must be described: are there any cases of mortality among bitten people who have not followed post-exposure treatment? Or, Does the hospital only give vaccination/treatment to people bitten with a confirmed link to animal rabies? Are all human bite cases linked to confirmed case of animal rabies?
If not, it is important to include the variable on the status of the animal involved if available (confirmed rabies or not confirmed). Are all 14 943 and 18 998 cases rabies-related, or are some suspected cases treated to avoid mortality? It is important to have this description. Even though the subject deals with bites, it's important to give a description of cases of bites with rabies. It's an epidemiological part, but it's important even if it's a general description in relation to the number given.

Response: We have addressed this in the revised manuscript. Please refer to line numbers 307-325.
